# Inferring the Demographic History of African Farmers and Pygmy Hunter–Gatherers Using a Multilocus Resequencing Data Set

**DOI:** 10.1371/journal.pgen.1000448

**Published:** 2009-04-10

**Authors:** Etienne Patin, Guillaume Laval, Luis B. Barreiro, Antonio Salas, Ornella Semino, Silvana Santachiara-Benerecetti, Kenneth K. Kidd, Judith R. Kidd, Lolke Van der Veen, Jean-Marie Hombert, Antoine Gessain, Alain Froment, Serge Bahuchet, Evelyne Heyer, Lluís Quintana-Murci

**Affiliations:** 1 Institut Pasteur, Human Evolutionary Genetics, CNRS, URA3012, Paris, France; 2 Unité d'Eco-Anthropologie et Ethnobiologie, MNHN/P7/CNRS UMR5145, Musée de l'Homme, Paris, France; 3 Unidade de Xenética, Instituto de Medicina Legal, Universidad de Santiago de Compostela, Galicia, Spain; 4 Dipartimento di Genetica e Microbiologia, Universita di Pavia, Pavia, Italy; 5 Department of Genetics, Yale University School of Medicine, New Haven, Connecticut, United States of America; 6 Laboratoire Dynamique Du Langage, CNRS UMR5596, Université Lumière Lyon 2, Lyon, France; 7 Unité d'Epidémiologie et Physiopathologie des Virus Oncogènes, Institut Pasteur, Paris, France; University of Chicago, United States of America

## Abstract

The transition from hunting and gathering to farming involved a major cultural innovation that has spread rapidly over most of the globe in the last ten millennia. In sub-Saharan Africa, hunter–gatherers have begun to shift toward an agriculture-based lifestyle over the last 5,000 years. Only a few populations still base their mode of subsistence on hunting and gathering. The Pygmies are considered to be the largest group of mobile hunter–gatherers of Africa. They dwell in equatorial rainforests and are characterized by their short mean stature. However, little is known about the chronology of the demographic events—size changes, population splits, and gene flow—ultimately giving rise to contemporary Pygmy (Western and Eastern) groups and neighboring agricultural populations. We studied the branching history of Pygmy hunter–gatherers and agricultural populations from Africa and estimated separation times and gene flow between these populations. We resequenced 24 independent noncoding regions across the genome, corresponding to a total of ∼33 kb per individual, in 236 samples from seven Pygmy and five agricultural populations dispersed over the African continent. We used simulation-based inference to identify the historical model best fitting our data. The model identified included the early divergence of the ancestors of Pygmy hunter–gatherers and farming populations ∼60,000 years ago, followed by a split of the Pygmies' ancestors into the Western and Eastern Pygmy groups ∼20,000 years ago. Our findings increase knowledge of the history of the peopling of the African continent in a region lacking archaeological data. An appreciation of the demographic and adaptive history of African populations with different modes of subsistence should improve our understanding of the influence of human lifestyles on genome diversity.

## Author Summary

The central African belt represents a key region for understanding recent changes in human history and modes of subsistence because the largest group of hunter–gatherers of Africa, the Pygmies, still inhabits this region and coexists with neighboring agricultural populations. However, the understanding of the peopling history of equatorial Africa is hampered by the rapid disintegration of fossil remains in the rainforest's acidic soils. When archaeology fails, population genetics can reconstruct the history of populations from their present-day genetic variation. We generated a large resequencing dataset in different farming, Western Pygmy, and Eastern Pygmy populations dispersed over the African continent. By means of simulation-based inferences, we show that the ancestors of Pygmy hunter–gatherers and farming populations started to diverge ∼60,000 years ago. This indicates that the transition to agriculture—occurring in Africa ∼5,000 years ago—was not responsible for the separation of the ancestors of modern-day Pygmies and farmers. We also show that Western and Eastern Pygmy groups separated roughly 20,000 years ago from a common ancestral population. This finding suggests that the shared physical and cultural features of Pygmies were inherited from a common ancestor, rather than reflecting convergent adaptation to the rainforest.

## Introduction

There is archaeological and genetic evidence to suggest that anatomically modern humans originated in a small, isolated population in Africa 150–200 thousand years ago (Kya). Worldwide population radiation then occurred 50–75 Kya [1]–[17]. However, the history of sub-Saharan African populations, which display considerable cultural, linguistic, phenotypic and genetic diversity, remains less clear [18],[19]. Studies based on multidisciplinary approaches generally indicate that sub-Saharan Africa was re-peopled recently, during the so-called Bantu expansions, extending outwards from a Nigeria-Cameroon homeland and beginning 3–5 Kya. These expansions were accompanied by the spread of Bantu languages, agricultural practices and sedentism, and probably also by iron working [20]–[23]. Most sub-Saharan African populations have now integrated these sociocultural practices, speaking one of the 450 Bantu languages [24] and presenting principally an agriculture-based sedentary lifestyle. However, a few populations did not adopt the lifestyle associated with Bantu expansions and continue to live as mobile groups, with a mode of subsistence based essentially on hunting and gathering. Today, these groups include the Western (e.g., Aka, Baka, Bakola) and Eastern (e.g., Efe, Asua, Sua) Pygmies, the Khoi, the San, the Okiek and the Hadza [25].

The Pygmy populations occupy a vast territory extending west-to-east along the central African belt from the Congo Basin to Lake Victoria. They have a mostly forest-dwelling hunter-gathering lifestyle, specific cultural practices (honey gathering tools, etc. [26]) and distinctive physical traits (e.g., lowest mean stature of all human populations [27],[28]). Pygmy groups traditionally live in huts, moving regularly from one camp to another, although some groups remain sedentary for some time due to socioeconomic dependence on neighboring farmers. Most Pygmy populations now speak the language of neighboring farming populations, suggesting extensive cultural — and possibly genetic — exchanges between the two groups [26], [27], [29]–[34]. Two main groups of Pygmy populations, each including different ethnic groups, are currently recognized: the “Western Pygmies” inhabiting the western part of the Central African rainforest corresponding broadly to the Congo Basin, and the “Eastern Pygmies” living in the easternmost part of the Central African belt close to the Ituri rainforest and Lake Victoria. Despite the extensive similarity in their modes of subsistence, cultural practices and distinctive phenotypic traits, Western and Eastern Pygmies clearly display both linguistic and genetic (at least for mtDNA and some protein markers) differentiation: the resemblance between each of the two Pygmy population groups and local farming populations is greater than that between the two Pygmy groups [8],[27],[35],[36].

Despite the large body of ethnological and linguistic data collected for these populations, little is known about the prehistory, population dynamics and past interactions between African farmers and Pygmy hunter-gatherers. Indeed, our understanding of the past peopling of Central Africa is limited by the virtual absence of human remains in its acidic soils [21]. In addition, the differences in the mode of subsistence of these two groups and the complex interactions between them raise several questions: which historical and demographic events led to the divergence between the ancestors of present-day farmers and Pygmies? Have the recent Bantu expansions associated with the spread of farming been responsible for the divergence of these two groups of populations? Or, were these populations already genetically — and possibly ecologically — differentiated before the agricultural revolution in Africa? How has the size of the populations of these two groups changed since they started to diverge? Furthermore, how did Western and Eastern Pygmy populations, which today show geographic separation, linguistic differentiation and distinctive genetic features, acquire their shared specific cultural and phenotypic traits? Did these two groups initially have a common ancestry but subsequently split apart, or do they reflect convergent cultural and genetic adaptation to the rainforest?

We addressed these questions by first considering the demographic characteristics of the agricultural, Western Pygmy, Eastern Pygmy population groups (i) to determine how these three population groups separated over time (i.e., branching order of the phylogenetic tree) and (ii) to estimate the time at which these population groups separated and the levels of subsequent gene flow between them. We generated a large multilocus resequencing dataset for five agricultural and seven Pygmy populations dispersed over the African continent. We then compared the ∼7.8 Mb of diploid sequences obtained with a large number of simulations exploring various demographic and branching scenarios, to identify the models best fitting the observed data. We then estimated, with the approximate Bayesian computation (ABC) method [37], population separation times and levels of gene flow between these populations under an isolation-with-migration (IM) framework — a realistic model assuming that populations diverge and subsequently experience gene flow. The model best fitting our data involves early divergence of the ancestors of farming populations and Pygmy hunter-gatherers ∼60,000 years ago, followed by a split of the Pygmies' ancestors into the Western and Eastern Pygmy groups ∼20,000 years ago. This study thus improves our understanding of the ancient history of the ecologically and culturally diverse populations of sub-Saharan Africa.

## Results/Discussion

**Figure 1:**
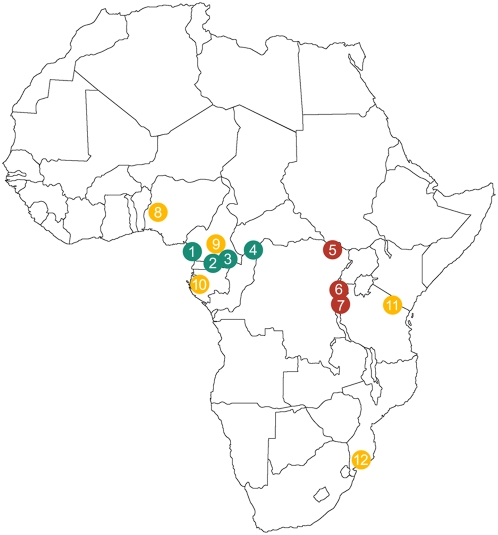
Geographic location of the 12 populations studied. Blue-green dots represent Western Pygmy (WPYG) populations, maroon dots represent Eastern Pygmy (EPYG) populations, and yellow dots represent agricultural (AGR) populations. 1. Bakola from Cameroon, 2. Baka from Gabon, 3. Baka from Cameroon, 4. Biaka from the Central Africa Republic, 5. Mbuti from the Democratic Republic of Congo, 6. Twa from northern Rwanda, 7. Twa from southern Rwanda, 8. Yoruba from Nigeria, 9. Ngumba from Cameroon, 10. Akele from Gabon, 11. Chagga from Tanzania, 12. Mozambicans from Mozambique.

To establish the branching history of agricultural, Western and Eastern Pygmy hunter–gatherer populations from Africa, and to coestimate separation times and levels of gene flow between these groups of populations, we resequenced 24 independent non coding genomic regions of ∼1.3 kb each, corresponding to a total of ∼33 kb per individual, including 20 autosomal regions, and one mtDNA, one Y-linked and two X-linked regions (Table S1). This resequencing was carried out in 236 individuals belonging to five different agricultural (AGR) populations, and four Western Pygmy (WPYG) and three Eastern Pygmy (EPYG) hunter-gatherer populations (Figure 1). As a first data quality filtering, we excluded samples presenting cryptic relatedness, a particularly common situation in traditional populations, because this can affect demographic inference [38]. Out of the 236 individuals, we excluded 20 individuals who appeared to be related on the basis of their genotypes, using the RELPAIR program [39] (Materials and Methods). In the resulting set of 216 unrelated samples, we identified a total of 413 SNPs, including 340 autosomal, 15 X-linked, 10 Y-linked and 48 mtDNA SNPs.

### Population Subdivision among Farmers and Pygmy Hunter–Gatherers

**Figure 2:**
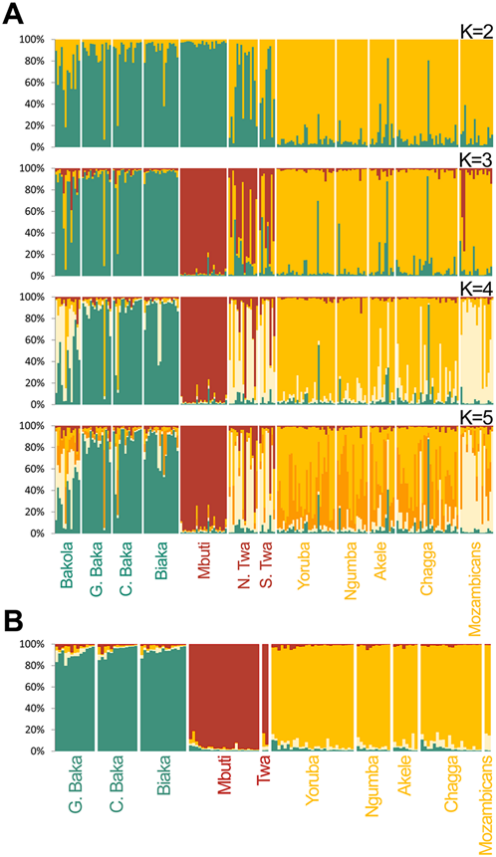
Estimated structure of populations of African farmers and Pygmy hunter–gatherers, based on autosomal and X-linked regions. Individuals are represented as thin vertical lines partitioned into segments corresponding to their membership of the genetic clusters indicated by the colors. G. and C. Baka stand for Gabonese and Cameroonese Baka, and N. Twa and S. Twa stand for Twa Pygmies from north and south of Rwanda, respectively. (A) Estimated structure of the entire population dataset, which includes all individuals except those displaying cryptic relatedness. *K*, the prior number of groups, varied from 2 (upper chart) to 5 (lower chart). For the models in which *K* was at least 5, the STRUCTURE program detected no additional cluster. The likelihood of the data was maximal at *K* = 4 (the mean ln[likelihood] values for *K* = 2, 3, 4 and 5 were equal to −16606, −16563, −16277 and −16290, respectively). (B) Estimated structure of the “filtered population dataset.” We excluded from this dataset those individuals whose proportion of ancestry in another population group was higher than 20% at *K* = 4, the most probable value of *K*. Using this filtering procedure, we excluded 92 individuals, including 15 Bakola, 2 C. Baka, 2 G. Baka, 4 Biaka, 1 Mbuti, and 21 Twa Pygmies, as well as 4 Yoruba, 5 Ngumba, 5 Akele, 12 Chagga, and 21 Mozambican farmers.

We first investigated whether our sampled populations constituted different genetic entities, by clustering individuals as a function of their genotypes for all autosomal and X-linked regions, using the STRUCTURE program [40]. When we specified that the data corresponded to only two groups (*K* = 2), Pygmy groups and AGR populations were separated into two different clusters (Figure 2A). This suggests that the genetic structure of African agricultural and Pygmy populations is correlated with their modes of subsistence. However, WPYG and EPYG groups further separated into two clusters at *K* = 3, revealing a certain degree of genetic differentiation between the two groups of Pygmy populations. The model with four clusters, which is the most probable given the data (P(*K* = 4/data) = 75.8%), further partitioned the group of farmers into those inhabiting the Central African belt and those located in South-East Africa. No other cluster was found for *K* values higher than 4 (Figure 2A). Overall, our results indicated that the three ethnologically recognized population groups — agricultural populations, Western Pygmies and Eastern Pygmies — corresponded broadly to different genetic entities.

However, STRUCTURE analysis revealed that some of the populations within each of these three population groups displayed considerable admixture or genetic differentiation (Figure 2A). Regardless of the value of *K* considered, three populations had large proportions of individuals with multiple memberships: the Bakola Pygmies from Cameroon and the two populations of Twa Pygmies from Rwanda. This observation confirms the admixed status of the Bakola Pygmies inferred from 28 autosomal microsatellites [41], indicating substantial levels of gene flow from neighboring farmers. With respect to the two populations of Twa Pygmies, they clearly clustered with South-East African farmers for *K* = 4, consistent with these Pygmy groups being admixed (some anthropologists describe them as “Pygmoids”), and with the complete shifting of their cultural practices towards those of neighboring agricultural populations [27]. Furthermore, the STRUCTURE analysis for *K* = 4 separated Mozambicans from the other agricultural populations (Figure 2A). This suggests the presence of fine-scale population structure in the AGR group, despite the very low and non significant levels of differentiation between AGR populations, on the basis of the *F*_ST_ statistics (Table S2).

Admixture or fine-scale population structure within each of our three population groups (i.e., AGR, WPYG and EPYG) may affect historical and demographic inferences [42]. We therefore conducted all subsequent analyses on a pruned population dataset. This “filtered population dataset” excludes individuals with ancestry in other populations, or populations that appear to be differentiated at *K* = 4 within each population group. The excluded individuals mostly corresponded to Bakola Pygmies, Twa Pygmies and Mozambicans (Figure 2B, Text S1). Only the results obtained with this filtered population dataset are discussed. However, we explored the effect of this filtering on our inferences, by also carrying out all analyses with the entire population dataset (the “composite population dataset”), which includes the admixed/structured individuals/populations (Text S1, Figure S1, S2, and S3, Tables S3, S4, S5, S6, and S7).

### Demographic Characteristics of African Farmers and Pygmy Hunter–Gatherers

**Figure 3:**
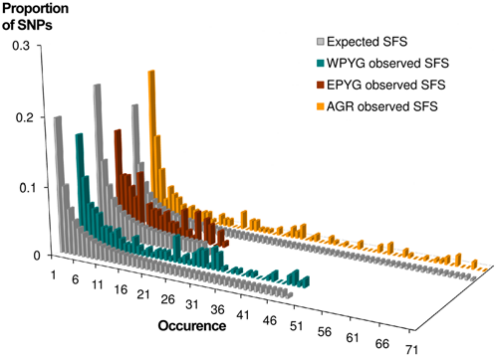
Site frequency spectra of the WPYG, EPYG, and AGR populations for the 20 autosomal regions, using the filtered population dataset. Gray histograms represent the expected site frequency spectra (SFS) of a constant-sized panmictic population with the same number of individuals as observed in the three population groups.

As departures from nonequilibrium demography (e.g., population growth or shrinkage) have been shown to influence the estimation of population separation times and levels of gene flow [43],[44], we first assessed the demographic history of each population group (AGR, WPYG and EPYG): we determined the simplest demographic model best fitting the observed within-population variation data for each population group, using a number of diversity indices and neutrality statistics summarizing the data (Table 1). The patterns of variation observed within the AGR group were characterized chiefly by an excess of low-frequency variants (Figure 3), as attested by the significant negative values obtained for some neutrality tests for autosomes and mtDNA (Table 1). The variance of the Tajima's *D* statistic was also significantly lower across autosomal regions in the AGR group (Table S4), this pattern being a specific signature of population growth [45]. These observations suggest the occurrence of population growth among the ancestors of present-day farmers. As all the farming populations studied here speak Benue-Congo languages (including Bantu languages), the signatures of population expansion and the low levels of differentiation (Table S2) observed among AGR populations may result from Bantu expansions spreading the farming lifestyle throughout sub-Saharan Africa over the last ∼5 Kya [20]–[23].

**Table 1:** Mean diversity indices and neutrality tests across the 24 independent genomic regions sequenced in the filtered population dataset of Western Pygmies (WPYG), Eastern Pygmies (EPYG), and African farmers (AGR).

		*S*	*π*	*θ*_W_	T*D* a ^,^ b	D* a ^,^ b	*Fs* a ^,^ b
**Twenty autosomal regions**	WPYG	159	0.00124	0.00117	0.045	0.076	−0.391
	EPYG	132	0.00126	0.00112	0.318	0.384	0.314
	AGR	192	0.00113	0.00131	**−0.428**	−0.121	**−1.404**
**Two X-linked regions**	WPYG	7	0.00076	0.00057	0.94193	0.94607	−0.1895
	EPYG	7	0.00066	0.0006	0.26186	0.33353	−0.0595
	AGR	9	0.00084	0.00066	1.02237	0.35796	0.8515
**One Y-linked region**	WPYG	4	0.00039	0.00042	−0.18504	−0.95131	−0.813
	EPYG	2	0.0003	0.00031	−0.06382	−0.22104	−0.239
	AGR	1	0.00006	0.00009	−0.42886	0.54491	−0.058
**One mtDNA region**	WPYG	16	0.00197	0.00312	−1.18262	−1.72393	−3.392
	EPYG	14	0.00317	0.00309	0.08644	0.72882	−6.329
	AGR	37	0.00289	0.00644	−1.78988	−1.79533	**−44.181**

a T*D*: Tajima's *D*; D*: Fu and Li's D*; *Fs*: Fu's *Fs*.

b Neutrality statistics in bold are statistically significant at the 5% level for all tests, except Fu's *Fs* (set at 2%). Variances of neutrality statistics across autosomal regions are reported in Table S4.

**Figure 4:**
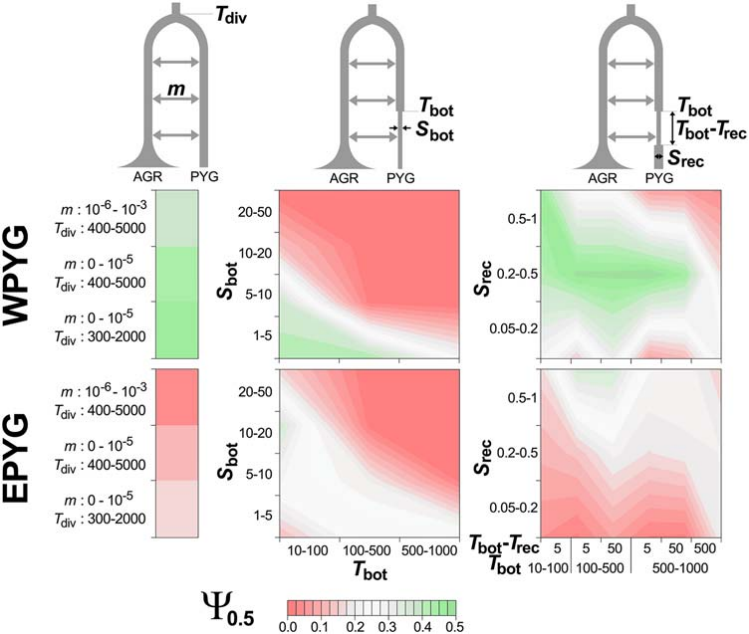
Different models simulating the demographic regime of the WPYG and EPYG groups and the mean proportion of small distances (*Ψ*_0.5_) obtained in comparisons with simulated statistics. Times are in generations. *T*_bot_ and *S*_bot_ are the time and strength of the bottleneck, respectively. *T*_rec_ and *S*_rec_ are the time and strength of the population-size recovery, respectively. Modeling details and the prior distributions of parameters are given in Table S8. We calculated the mean *Ψ*_0.5_ for a given model and set of parameters, by resampling, among 100,000 simulations, 100 sets of 10,000 simulations of the model, calculating *Ψ*_0.5_ for each set and reporting the mean *Ψ*_0.5_ across sets. The model with one bottleneck (*T*_bot_: 100–1000 generations, *S*_bot_ = 5) and one recovery (*T*_rec_ = *T*_bot_-5 generations, *S*_rec_: 0.2–0.5) generated, for the WPYG group, the maximum *Ψ*_0.5_ in 76% of cases when compared with all models, and in 96% of cases when compared with only constant population-size models. For the EPYG group, the model with one bottleneck (*T*_bot_: 10–100 generations, *S*_bot_ = 10–20) generated the maximum *Ψ*_0.5_ in 28% of cases when compared with all models, and in 100% of cases when compared only with constant population-size models.

None of the classical neutrality tests used detected significant departures from the constant-sized population model for the WPYG and EPYG groups (Table 1, Figure 3). However, the occurrence of gene flow between populations with different demographic regimes may dilute the signals of departure from nonequilibrium demography detected by neutrality tests (e.g., the signature of a bottleneck among Pygmies is erased by gene flow with the expanding AGR populations, introducing low-frequency variants into the Pygmy gene pool). We identified the demographic model best fitting the Pygmy data by comparing the within-population summary statistics of WPYG and EPYG (Table 1) with simulated summary statistics under constant-population size and bottleneck models, in the presence of various levels of gene flow with an expanding AGR population (Figure 4, Table S8, Materials and Methods for details). The genetic diversity of both WPYG and EPYG fitted significantly better with models assuming a bottleneck in the Pygmy population accompanied by high levels of gene flow with the AGR population than with a model of a constant-sized Pygmy population with negligible gene flow with the AGR population. A bottleneck beginning 2,500–25,000 years ago with an 80% decrease in population size, followed by a recovery starting 125 years later with a size increase of between 100% and 400% (Figure 4), fitted the WPYG data significantly better than the constant-sized population model (*P* = 0.04, see Materials and Methods). For the EPYG group, a bottleneck starting 250–2,500 years ago with a 90 to 95% decrease in population size (Figure 4) fitted the observed genetic diversity significantly better than the constant-sized population model (*P*<0.01). Population structure models could also theoretically fit the PYG data, in the presence of gene flow with AGR populations. However, the occurrence of population structure in PYG populations alone is unlikely because (i) our analyses considered a pruned population dataset excluding admixed populations (Figure 2B) and (ii) the influence of population structure within WPYG populations is probably negligible because within-population neutrality statistics for each WPYG population individually were always positive (Text S1). Altogether, our adjustment for the demographic regime of each population group revealed the occurrence of population growth in AGR populations and bottlenecks in both the WPYG and EPYG groups.

### The Branching Model: Autosomal Evidence of a Recent Common Origin of the Western and Eastern Pygmy Groups

**Figure 5:**
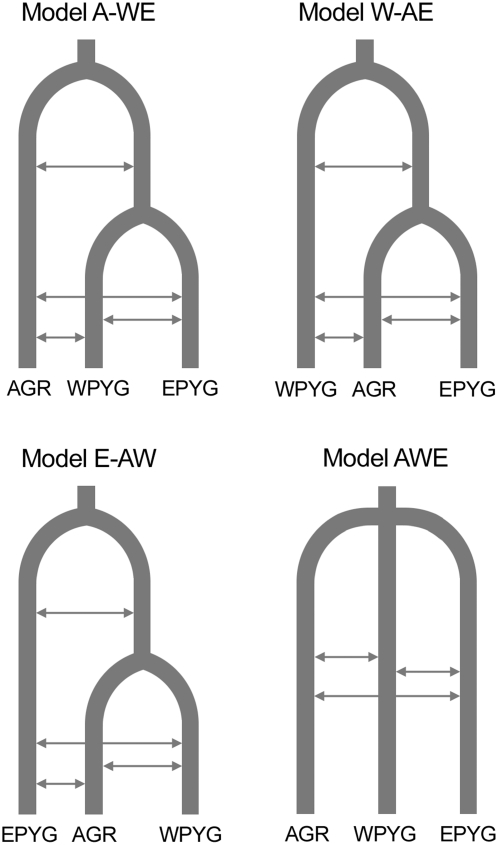
Four possible models explaining the branching history of African farmers, Western Pygmies, and Eastern Pygmies. Arrows indicate symmetric gene flow.

The sequence of the divergence events underlying the current differentiation of Western Pygmy, Eastern Pygmy and agricultural groups remains unclear. All Pygmy groups share idiosyncratic cultural and phenotypic traits, but substantial linguistic and genetic differentiation between Pygmy groups is also observed [8],[27],[35],[36],[46]. These observations call into question the postulated common origin of African “Pygmy” populations. Indeed, if Western and Eastern Pygmy groups share a more recent ancestry with their respective agricultural neighbors than with each other, then they may have acquired their shared specific traits by convergence rather than by shared ancestry. Various models can be put forward to explain the current levels of differentiation between these three different groups: (i) the *A-WE* model, involving an ancient divergence between the ancestors of the AGR and PYG groups, followed by a split of PYG ancestors into the WPYG and EPYG groups; (ii) the *W-AE* model, in which the most ancient split is that between the ancestors of the WPYG and AGR groups; (iii) the *E-AW* model, in which the most ancient divergence is that between the ancestors of the EPYG and AGR groups, and (iv) the *AWE* model, in which all populations diverged simultaneously (Figure 5). To discriminate between these four models, we calculated several between-population summary statistics for all pairs of populations, including *F*_ST_, the proportion of shared mutations, the proportion of low-frequency shared mutations, and the mean frequency of shared mutations (Table S5, Materials and Methods)

**Figure 6:**
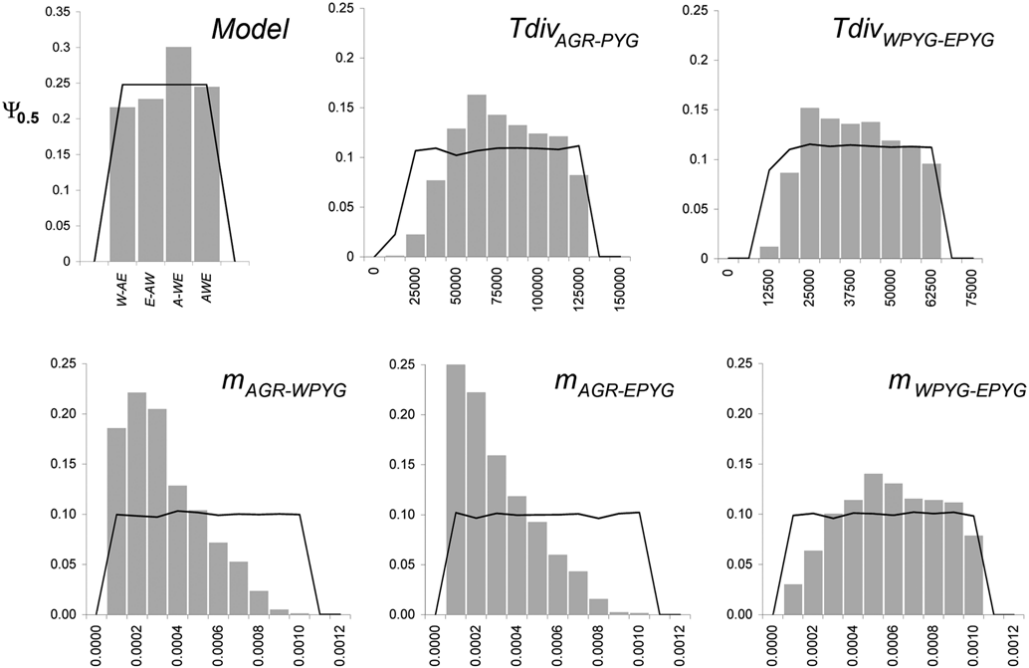
Prior and approximated posterior distributions of the IM model and IM parameters under the best-fit *A-WE* model. Black lines represent prior distributions and gray histograms represent approximated posterior distributions obtained by the ABC method [37], except for model choice, for which the posterior distribution was estimated based on the proportions of small distances generated by each model (see Materials and Methods). Divergence times *Tdiv* are expressed in years and migration rates *m* in proportion of migrants per generation. The prior and approximated posterior distributions of the IM model and IM parameters under the best-fit *A-WE* model were obtained using the filtered population dataset. Those obtained using the composite population dataset are reported in Figure S3. Of note, the posterior distributions obtained with the composite population dataset were generally more narrowly peaked than those obtained with the filtered population dataset.

Twenty autosomal regions were simulated 1,000,000 times under the four possible IM models (Figure 5) with IM parameters (times of divergence, migration rates) drawn from large, flat prior distributions (Table S9). As the specific demographic history of each population group may influence the inference of the branching history, we incorporated into our simulations (Table S9) the demographic model identified for each population group most compatible with their observed within-population summary statistics (Table 1). The mean between-population summary statistics across the 20 simulated regions were then compared with the observed statistics for the 20 autosomal regions (Table S5, Materials and Methods). The proportion of low-frequency shared mutations and the mean frequency of shared mutations were found to be non informative: their mean values were similar across the four IM models simulated (data not shown). By contrast, *F*_ST_ and the proportion of shared mutations varied considerably between IM models. These two statistics were therefore systematically considered in the sets of summary statistics used for the best-fit approach (Materials and Methods). Independently of the set of summary statistics used, the *A-WE* model always gave the highest proportion of small distances between the simulated and observed datasets (*Ψ*_0.5_), and was therefore identified as the most probable model given the data (Figure 6). We then investigated whether this result was sensitive to *ξ* — the threshold at which distances between simulated and observed statistics are considered to be “small” (Materials and Methods). We observed a highly significant negative correlation between *ξ* and the proportion of small distances *Ψ_ξ_* generated by the *A-WE* model (*r^2^* = 0.969, *P* = 0.00014): the smaller *ξ*, the better the simulations fitted the observed data, and the greater the enrichment of the *A-WE* model in these simulations. This analysis thus clearly supports our conclusion that the *A-WE* model is the most probable, given the autosomal data.

Unlike autosomal, X-linked and Y-linked regions, mtDNA displayed strong differentiation between Western and Eastern Pygmies (Table S5), an observation at odds with the *A-WE* model. Several lines of evidence suggest that sex-biased gene flow, ancient maternal population structure and/or stronger genetic drift have contributed to the high levels of mtDNA differentiation observed today between the two Pygmy groups (Text S1 for details). More generally, genetic drift has probably been greater among PYG populations for all genomic compartments, because the PYG *N*_e_ is smaller than the AGR *N*_e_, potentially leading to higher levels of differentiation between the two PYG groups than between each PYG group and the AGR group. Indeed, when simulating the 20 autosomal regions under the *AWE* model, in which the three populations diverge simultaneously, greater mean differentiation was observed between the two PYG groups than between the PYG and AGR populations (data not shown). Consequently, a more recent divergence between the two Pygmy groups (than between the PYG and AGR groups) is required, both to compensate for the stronger genetic drift among PYG populations and to generate the observed lower level of differentiation of autosomal regions between the two PYG groups. Taken together, our analyses, which explored a wide range of models and parameter values (Table S9), clearly support the hypothesis of a recent common origin of Western and Eastern African Pygmies.

### Estimates of Population Separation Times and Levels of Gene Flow: An Approximate Bayesian Computation Approach

We then investigated the time scale of the various events characterizing the branching history of AGR, WPYG and EPYG populations, by estimating IM parameters under the validated *A-WE* model. The coestimation of population separation time and gene flow levels between two populations is generally difficult because low levels of differentiation may result from either a recent splitting of populations with low subsequent gene flow or from an ancient split with high subsequent gene flow [47]. Several methods have been developed for confident estimation of IM parameters, provided that some fixed differences between diverging groups are observed (i.e., species or subspecies) [48]–[50]. These methods are also limited to an IM model with only two populations, or to constant-sized populations. The application of two of these methods to our dataset — IMa and *mimar* [49],[50] — provided no evidence of chain convergence despite good mixing of the Markov chains (Text S1), probably due to the low overall levels of differentiation between the PYG and AGR groups (i.e., no fixed differences observed between the two groups). We therefore sought to coestimate these parameters under the ABC framework [37]. We obtained non-flat unimodal posterior distributions for all IM parameters (Figure 6), using different informative summary statistics (Materials and Methods). We assessed the accuracy of these estimations, by estimating IM parameters for randomly chosen simulations as if they were empirical data, but with known actual parameter values. In ∼95% of cases, the known parameter values were within the 95% confidence interval of parameter estimates (Table 2), indicating that estimated confidence intervals were accurate.

**Table 2:** Estimates, confidence intervals, and accuracy of estimations of population separation times and levels of gene flow between WPYG, EPYG, and AGR groups, under the most probable *A-WE* model.

		Estimate	95% Confidence Interval (CI)	Accuracy
**Filtered population dataset**	*N_A_*	11,402	[7,670–15,653]	96%
	*Tdiv_AGR-PYG_*	56,049	[25,814–130,548]	94%
	*Tdiv_WPYG-EPYG_*	21,903	[14,218–66,313]	98%
	*m_AGR-WPYG_*	1.76×10^−4^	[0–7.04×10^−4^]	97%
	*m_AGR-EPYG_*	2.38×10^−5^	[0–6.73×10^−4^]	97%
	*m_WPYG-EPYG_*	4.42×10^−4^	[7.46×10^−5^–1.03×10^−3^]	97%
**Composite population dataset**	*N_A_*	9,428	[6,791–15,151]	96%
	*Tdiv_AGR-PYG_*	60,061	[25,240–120,091]	95%
	*Tdiv_WPYG-EPYG_*	24,583	[10,082–62,365]	94%
	*m_AGR-WPYG_*	2.15×10^−4^	[0–6.96×10^−4^]	98%
	*m_AGR-EPYG_*	6.63×10^−5^	[0–5.34×10^−4^]	95%
	*m_WPYG-EPYG_*	5.79×10^−4^	[1.34×10^−4^–1.02×10^−3^]	97%

The ancestral population size *N_A_* is given in individuals, population separation times *Tdiv* in years, and levels of gene flow *m* in proportion of migrants per generation. Estimates correspond to the mode of posterior distributions (Figures 6 and S3). The accuracy of estimation was assessed by estimating IM parameters of simulations with known parameter values. The percentage is the proportion of known parameter values that fall into the estimated 95% CI.

Our estimations indicated that the ancestral effective population size of the African groups here studied was 11,402 individuals (95% CI: 7,670–15,653) (Table 2). This ancestral population pool started to diverge, eventually generating the current agricultural and Pygmy populations, 56 Kya (95% CI: 25.8–130.5). The subsequent split of the ancestors of Pygmies into the present-day WPYG and EPYG groups was estimated at 21.9 Kya (95%CI: 14.2–66.3). Finally, our estimates for the levels of gene flow between WPYG and EPYG, between WPYG and AGR and between EPYG and AGR populations were 4.4×10^−4^, 1.8×10^−4^ and 2.4×10^−5^, respectively.

As previously mentioned, all analyses (adjustment of the internal demographic regimes of each population group, the branching model and ABC estimation of IM parameters) were also performed with the “composite population dataset”, which includes the admixed/structured individuals/populations (Text S1, Figures S1, S2, and S3, Tables S3, S4, S5, S6, and S7). The results obtained for this entire-population dataset were remarkably similar to those obtained with the pruned population dataset: the best-fitting branching model of populations was the same (i.e., the *A-WE* model, Figure S3) and the estimates of population separation times were very similar (Table 2, Figure S3). However, estimates of gene flow between population groups were consistently lower for the filtered population dataset, which excludes admixed individuals/populations. Thus, the pooling of populations with different proportions of admixed individuals had no effect on the estimation of population separation times. This highlights the reliability of the ABC approach for estimating population divergence by properly adjusting for the different levels of gene flow between populations.

### Implications for African Prehistory

The implications of our estimates are important for broader issues in African prehistory, although they must be interpreted carefully because of their large confidence intervals (Table 2). The finding that the ancestors of AGR and PYG populations diverged *ca*. 60 Kya is consistent with our recent single-locus estimation based on the mtDNA diversity of African farmers and Western Pygmies [36]. Most of the large waves of population expansion and migration, both within and out of Africa, have been dated at *ca*. 50–80 Kya, based on several genetic markers [1]–[17]. It has been suggested that these early population movements within and out of Africa may have been triggered by rapid environmental changes. During this period, sub-Saharan Africa witnessed a major episode of climatic change: a sharp oscillation towards a drier climate, with annual rainfall decreasing by up to 50% [51]. These early population expansions may also have been fuelled by increases in the carrying capacity of some human groups associated with radical changes in technology with the emergence of more complex hunting equipment and large-scale movements of high-quality stone and imported shell ornaments [16]. The environmental changes occurring at this time therefore seem to have favored a drastic increase in the complexity of the technological, economic, and social behavior of certain African groups, potentially leading to a major demographic expansion of these groups in competition with other, adjacent groups [16]. In this context, our estimated date of the initial divergence between the ancestors of present-day farmers and Pygmies implies that this period was characterized not only by major human movements, but also by early episodes of population differentiation within the African continent.

Our evidence for a separation of the ancestors of Western and Eastern Pygmy groups *ca*. 20 Kya is also consistent with a previous mtDNA study, dating the time of separation of these two Pygmy groups to at least 18 Kya [52]. These estimates coincide with another period of major climatic change, the Last Glacial Maximum, which led to a massive retreat of tropical forests in Central Africa [53]–[55]. Our genetic results thus support the anthropological hypothesis that the ancestors of present-day forest specialists — Western and Eastern Pygmies — began to diverge at the same time as the rainforest retreated into refugia, ∼20 Kya [26]. However, the split of Pygmy populations into two pockets corresponding to forest refugia did not totally prevent the occurrence of gene flow between Western and Eastern Pygmy groups (Table 2). Finally, our estimates of gene flow between each group of Pygmies and agricultural populations yielded contrasting values, with levels of gene flow between WPYG and AGR populations three to seven times higher than those between EPYG and AGR populations (Table 2). This result, together with those obtained with protein markers [27], mtDNA [8],[36] and autosomal microsatellites [41],[46], indicates that (i) substantial gene flow has occurred between Western Pygmies and agricultural populations, possibly during a period before the strong social barriers currently separating these two groups became established [29],[32],[33],[41],[56], and (ii) the Eastern Mbuti Pygmies (i.e., the EPYG group in the filtered population dataset) have probably been among the most isolated Pygmy populations of sub-Saharan Africa.

### Conclusion

Our multilocus resequencing analyses, supported by simulation-based inferences, increase our knowledge of the peopling history of the African continent by revealing that: (i) Western and Eastern Pygmies share a recent common ancestry, indicating that their shared specific traits, such as hunting and gathering in rainforest ecosystems and short stature, were acquired by shared ancestry rather than by convergence, and (ii) the agricultural revolution associated with Bantu expansions is not responsible for the population differentiation currently observed between farmers and Pygmy hunter-gatherers, suggesting that the ancestors of these two populations had a hunting and gathering lifestyle but possibly in different, specific ecological habitats (e.g., forest and savanna). The distribution of lithic industries in the Middle Stone Age points to the existence of hunter-gathering groups in the open savanna environment of Central Africa [21]. This, together with the observation that Bantu migrations followed savanna passages [21], supports the notion that the mode of subsistence of the ancestors of farmers was savanna-based hunting and gathering.

The null model of selective neutrality provided by this study will also prove useful for the identification of genetic variants contributing to complex diseases and for the detection of genomic regions targeted by natural selection. In particular, a detailed study of the genome-wide footprints of local positive selection in African farmers and Pygmy hunter-gatherers, integrating the demographic model determined in this study, should facilitate robust identification of the population-specific adaptive responses of these two human groups to their different climatic, pathogenic and nutritional environments. These studies should help to decipher the potential genetic basis of the population-specific traits characterizing these ethnic groups, such as the short mean stature of the Pygmies. More generally, an appreciation of the demographic and adaptive history of these populations will improve our understanding of the influence of human lifestyles on genome diversity in terms of both health and disease.

## Materials and Methods

### DNA Samples

Sequence variation was surveyed in DNA samples from 12 sub-Saharan African populations. The panel included 118 samples from five agricultural populations (Yoruba from Nigeria [N = 31], Ngumba from Cameroon [N = 16], Akele from Gabon [N = 16], Chagga from Tanzania [N = 32] and Mozambicans [N = 23]), 71 samples from four Western Pygmy populations (Bakola from Cameroon [N = 16], Baka from Cameroon [N = 15], Baka from Gabon [N = 16] and Biaka from the Central Africa Republic [N = 24]), and 47 samples from three Eastern Pygmy populations (Mbuti from the Democratic Republic of Congo [N = 24] and Twa from southern [N = 8] and northern [N = 15] Rwanda) (Figure 1). The Biaka, Mbuti, Yoruba, and Chagga samples are subsets of samples described in ALFRED (http://alfred.med.yale.edu/alfred/index.asp) under sample UID numbers SA000005F, SA000006G, SA001805O, and SA000487T, respectively. All sampled individuals were healthy donors from whom informed consent was obtained. This study was approved by the Institut Pasteur Institutional Review Board (n° RBM 2008.06).

### Resequencing Dataset

The 24 independent regions sequenced here represent a total sequence length of 32.75 kb per individual (mean sequence length per region of 1.31 kb). We selected 20 non coding, independent autosomal regions (Table S1) to decipher the genetic architecture of AGR and PYG populations. The regions were selected (i) to be at least 200 kb away from any known or predicted gene or spliced EST (distance determined by inspection of the hg18 UCSC genome assembly); (ii) not to be in linkage disequilibrium (LD) with any known or predicted gene or spliced EST (as determined by inspection of the LD levels observed in the four HapMap populations, release 16); (iii) not to be in LD with each other and (iv) to have a region of homology with the chimpanzee genome (November 2003 release). We also selected two X-linked regions based on the same criteria, together with two linked regions on each arm of the Y chromosome and one mtDNA region selected in a previous study [57] (Table S1). The two Y-linked regions were considered as a single region in all analyses. All non coding regions were sequenced with two different primers. All sequencing reactions were run on automated capillary sequencers (ABI3130 and ABI3730). PCR and sequencing primers and protocols are available upon request. Samples from Mozambique and Rwanda underwent whole-genome amplification before PCR amplification and resequencing. Sequence alignment and SNP detection were carried out with Genalys v.3.3b [58]. In addition, all ABI base-called sequences and genotypes were visually inspected by two independent investigators. All singletons were confirmed by reamplification and resequencing. No false singleton was observed. Less than 0.1% of genotypes were left as missing data.

### Data Analysis

We reconstructed haplotypes with PHASE v.2.1 [59], using five independent runs with different seeds for each of the 22 recombining regions. For X-linked regions, we specified in PHASE that the phase of male haplotypes was known. All runs gave very similar reconstructions. Cryptic relatedness was assessed using the RELPAIR program v.2.0.1 [39]. We divided our population samples into two geographic areas: Western Africa (populations 1–4 and 8–10 in Figure 1) and Eastern Africa (populations 5–7 and 11–12 in Figure 1). We tested cryptic relatedness only between individuals coming from the same geographic area. We considered a pair of individuals as cryptically related when the likelihood of their inferred relationship was >1,000 higher than the likelihood of no cryptic relatedness between them. Twenty individuals were excluded based on this criterion: 1 G. Baka, 3 Bakola and 6 Biaka Pygmies, and 1 Yoruba, 3 Akele and 6 Mozambican farmers. Genetic membership of populations was inferred with STRUCTURE v.2.1 software [40], using the “correlations” and “admixture” models, with and without prior information about populations, 1,000,000 burn-in steps and 1,000,000 Monte Carlo Markov chain replications. We excluded the Y-linked and mtDNA regions from the STRUCTURE analysis because this program accepts only diploid loci. We recoded the 20 autosomal and two X-linked regions as microsatellites, considering each haplotype as an allele of a single multi-allelic locus. For each prior *K* value (2, 3, 4 and 5), we ran 20 independent runs with different seeds and found likelihoods to be stable across runs. We focused on several aspects of our resequencing dataset, including classical diversity indices (nucleotide diversity *π*, Watterson's estimator of theta *θ*_W_ and haplotype diversity *Hd*), neutrality statistics (Tajima's *D*, Fu & Li's D*, Fu's *Fs* and their mean and variance across regions) and population differentiation statistics (pairwise *F*_ST_). All these statistics, the observed site frequency spectra and those expected under a constant population size model, as well as the significance of *F*_ST_ values, were obtained with DnaSP v.4.10.9 [60]. Novel summary statistics were also developed to capture particular aspects of the genetic data: the proportion of shared mutations between populations 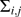, the proportion of low-frequency shared mutations 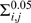 and the mean frequency of shared mutations 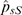, which were defined as follows. Consider *S* mutations segregating in populations *i* and *j*. Then 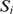 is the number of segregating sites in population *i*, 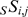 the number of segregating sites shared between populations *i* and *j* and 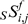 the number of shared segregating sites between populations *i* and *j* with a relative frequency in merged populations lower than *f*.

Then 
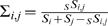
 and 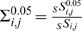.

### Coalescence Simulations

We used coalescence simulations (i) to assess the statistical significance of observed neutrality statistics and their means and variances across autosomal regions and (ii) to determine which models and parameters best fitted our empirical data. Simulations were performed using coalescent theory, as implemented in SIMCOAL v.2.1.2 [61], and using mutation rates (*μ*) and effective population sizes (*N*_e_) drawn from gamma distributions (Table S10), as in previous studies [17],[62]. The mean mutation rates of autosomal, X- and Y-linked regions were calculated from human-chimpanzee divergence, assuming that the two species diverged 6 million years ago [63] and a generation time of 25 years. For mtDNA, we used the synonymous mutation rate calculated in a previous study [14]. For all genomic regions, the number of mutations for the observed and simulated data was found to be similar (data not shown).

For each independent genomic region, the statistical significance of the neutrality statistics in each population group was assessed by comparing observed values with 100,000 values obtained from simulations of a sample, the size of which corresponded to that of the tested population sample, under a neutral model of evolution, assuming a constant population size and no recombination (only ∼0.5% of haplotypes at autosomal regions showed evidence of recombination). The statistical significance of means and variances of neutrality statistics across the 20 autosomal regions was assessed by simulating 100,000 sets of 20 independent regions under the same assumptions. Models were tested by simulating 100,000 and 250,000 datasets under each demographic and IM models respectively, with model parameters randomly drawn from prior distributions (see section below).

### Testing of Best-Fit Models

For both the adjustment of the demographic regimes of each population group and the assessment of the branching history of population groups, the simulated model that best fitted our autosomal data was defined as that giving the highest proportion of small distances (*Ψ_ξ_*) between the simulated and observed summary statistics, S′ and S. These distances were measured by calculating the normalized metric *D*(S′,S) [64], and *D*(S′,S) was considered to be small when lower than *ξ* = 0.5. This flexible statistical framework, which is based on comparisons between simulations and observed data, makes it possible to test complex models with fluctuations in effective population size, population separation times and gene flow, without estimating the real likelihood of the data (*ξ* = 0), which would be unfeasible given the complexity of the data and the models. The tested demographic and IM models were all simulated with prior distributions of model parameters (Tables S6, S7, S8, S9). We assessed whether a given model fitted the empirical data significantly better than another model, by resampling 100 times 10,000 simulations of each model, calculating for each model *Ψ*_0.5_ and estimating the *P*-value using a chi-square test comparing the proportion of small distances between the simulated and observed data, generated by each of the two models. The final *P*-value is the mean of the *P*-values obtained across the 100 resampling sets.

For all model testing procedures, only the autosomal dataset was considered. Before estimating levels of divergence and gene flow between populations, we determined a demographic scenario best accounting for the observed within-population summary statistics of our three population groups (AGR, WPYG and EPYG). We did not aim to identify a best-fitting model for the demographic regime of AGR populations, because historical [20],[21],[23], linguistic [22] and previous genetic studies [8],[36],[65],[66] strongly suggest that these populations have indeed undergone expansion. For our filtered population dataset of AGR individuals, we considered a single, recent population expansion, with the time of onset and exponential growth rate drawn from flat prior distributions (time of onset: 5–7.5 Kya; growth rate: 0.005–0.01). Simulated summary statistics (*S*, *π*, Tajima's *D* and Fu & Li's D*) under this demographic expansion were similar to the observed statistics for the AGR group (data not shown). For Pygmy populations, we compared the empirical summary statistics obtained for the WPYG and EPYG population groups (Table 1) with summary statistics for 3,000,000 simulations, considering 33 models of a constant-sized population or bottlenecks, varying in intensity, timing and duration (Figure 4, Table S8). We considered this population to have experienced varying levels of gene flow with an expanding population (Table S8) presenting mean summary statistics similar to those observed in the AGR population group (Table 1). The number of polymorphisms *S*, *π*, Tajima's *D* and Fu & Li's D* observed in the two PYG groups were chosen as the summary statistics for comparisons between simulated and observed data. This adjustment of the demographic regime of each population group was also performed for the composite population dataset (Text S1, Figure S2, Tables S3 and S6).

We then investigated the branching history of the three population groups (AGR, WPYG and EPYG), considering the previously described population-specific demographic models for each population group (Table S9): a model of a population expansion for AGR, a model of bottleneck with recovery for WPYG, and a model of bottleneck for EPYG. We tested four different models potentially accounting for the current genetic differentiation of the three population groups (Figure 5), using large flat prior distributions for separation time and migration rate parameters, except that the time of the oldest divergence was necessarily constrained by the time of the latest divergence (Table S9). We simulated 250,000 sets of 20 unlinked autosomal regions for each of the four IM models (Figure 5). We selected several summary statistics to discriminate between the confounding effects of divergence and gene flow on genetic variation: the proportion of mutations shared between populations 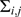, the proportion of low-frequency shared mutations 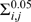, the mean frequency of shared mutations 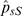, and pairwise *F*_ST_ (Text S1, Figure S5, Table S5). We tested several combinations of statistics summarizing the within- and between-population genetic diversity (data not shown). Finally, we used a set of statistics that included *S*, *π*, Tajima's *D*, Fu & Li's D* for each population group and pairwise *F*_ST_ and 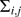 for each pair of population groups. This procedure (i.e., incorporation of the demographic characteristics of each population group into the estimation of their branching order) was also applied to the composite population dataset (Text S1, Figure S3, Tables S5 and S7).

### Parameter Estimation by Approximate Bayesian Computation (ABC)

Parameter estimation was based on the autosomal data alone. We estimated parameters under the best-fitting IM model (i.e., the *A-WE* model; Figure 6), by comparing our empirical data with 250,000 simulations of 20 independent regions under the *A-WE* model, using large flat prior distributions for separation time and migration rate parameters, except that the time of the oldest divergence was necessarily constrained by the time of the latest divergence (Table S9). We then used the ABC method, which generates posterior distributions of the parameters of interest deduced from parameter values of simulations satisfying the *D*(S′,S)<*ξ* criterion (see previous section and [37] for more details), with *ξ* chosen so that only 5,000 of 250,000 simulations are retained [17]. For the ABC procedure, we used the following summary statistics: *S*, *π*, Tajima's *D*, Fu & Li's D* for each population group and pairwise *F*_ST_ and 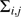 for each pair of population groups. This method was demonstrated to be accurate by estimating IM parameters for 100 simulated datasets for which the IM parameters were specified. Known parameter values were then compared with the 95% confidence interval (CI) for the ABC estimates of the parameter considered. Accuracy was estimated as the proportion of comparisons for which the known values were within the 95% CI for the estimated parameters. This procedure (i.e. ABC estimation of IM parameters) was also applied to the composite population dataset (Table 2, Text S1, Figure S3).

## Supporting Information

Figure S1 Site frequency spectra of the WPYG, EPYG and AGR populations for the 20 autosomal regions, using the composite population dataset. Gray histograms represent the expected SFS of a constant-sized panmictic population with the same number of individuals as observed in the three population groups. The right tail of the agricultural SFS has been truncated for clarity.
(8.29 MB TIF)

Figure S2 Different models simulating the demographic regime of the WPYG and EPYG groups and the mean proportion of small distances (*Ψ*_0.5_) obtained in comparisons with simulated statistics, based on the composite population dataset. Times are in generations. *T*_bot_ and *S*_bot_ are the time and strength of the bottleneck, respectively. *T*_rec_ and *S*_rec_ are the time and strength of the population size recovery, respectively. Modeling details and the prior distributions of parameters are given in Table S6. We calculated the mean *Ψ*_0.5_ for a given model and set of parameters, by resampling, among 100,000 simulations, 100 sets of 10,000 simulations of the model, calculating *Ψ*_0.5_ for each set and reporting the mean *Ψ*_0.5_ across sets. The model with one bottleneck (*T*_bot_: 10–100 generations, *S*_bot_ = 5) and one recovery (*T*_rec_ = *T*_bot_ - 5 generations, *S*_rec_: 0.5–1) generated, for WPYG, the maximum *Ψ*_0.5_ in 62% of cases when compared with all models and in 98% of cases when compared with only constant population size models. For the EPYG group, the constant population size model generated the maximum *Ψ*_0.5_ in 56% of cases when compared with all models.
(10.20 MB TIF)

Figure S3 Prior and approximated posterior distributions of the IM model and IM parameters under the best-fit *A-WE* model for the composite population dataset. Divergence times *Tdiv* are expressed in years and migration rates *m* in proportion of migrants per generation. Black lines represent prior distributions and gray histograms represent approximated posterior distributions obtained by the ABC method [37], except for model choice, for which the posterior distribution was estimated based on the proportions of small distances generated by each model (Materials and Methods). We observed a highly significant negative correlation between *ξ* - the threshold at which distances between simulated and observed statistics are considered to be “small” (Materials and Methods) - and the proportion of small distances *Ψ_ξ_* generated by the A-WE model (*r* ^2^ = 0.946, *P*<0.0001). The joint approximated posterior distribution of *Tdiv*_WPYG-EPYG_ and *m*_WPYG-EPYG_ is shown in Figure S4.
(6.07 MB TIF)

Figure S4 Approximated joint posterior distribution of the time of divergence and migration rate between Western and Eastern Pygmies for the composite population dataset. The posterior distribution of the two parameters is estimated by means of the proportion of small distances *Ψ*_0.5_. The time of divergence *Tdiv*_WPYG-EPYG_ and the migration rate *m*_WPYG-EPYG_ are reported in generations and in proportion of migrants per generation, respectively.
(4.52 MB TIF)

Figure S5 Behavior of selected summary statistics under various levels of divergence and gene flow. Time of divergence (in generations) and migration rate (in proportion of migrants per generation) are represented by *Tdiv* and *m*, respectively.
(4.93 MB TIF)

Table S1 Location of the 25 resequenced regions and their respective distances to coding regions.
(0.09 MB DOC)

Table S2 Mean pairwise *F*_ST_ values among the 12 sub-Saharan African populations for (A) 20 autosomal regions, (B) two X regions, (C) one Y region and (D) one mtDNA region.
(0.14 MB DOC)

Table S3 Mean diversity indices and neutrality tests across the 24 independent genomic regions sequenced in the composite population dataset of WPYG, EPYG and AGR.
(0.05 MB DOC)

Table S4 Variances of statistics from sequence-based neutrality tests across the 20 autosomal regions in WPYG, EPYG and AGR populations, using the filtered and composite population datasets.
(0.03 MB DOC)

Table S5 Mean summary statistics for genetic differentiation between the WPYG, EPYG and AGR populations, across the 24 genomic regions, for the filtered and composite population datasets.
(0.06 MB DOC)

Table S6 Prior distributions of the parameters of 33 models simulated to assess the demographic regime of Pygmy population groups, using the composite population dataset.
(0.12 MB DOC)

Table S7 Prior distributions of the parameters of the IM models simulated to assess the branching history of the AGR, WPYG and EPYG populations, using the composite population dataset.
(0.04 MB DOC)

Table S8 Prior distributions of the parameters of 33 models simulated to assess the demographic regime of Pygmy population groups, using the filtered population dataset.
(0.11 MB DOC)

Table S9 Prior distributions of the parameters of the IM models simulated to assess the branching history of the AGR, WPYG and EPYG populations, using the filtered population dataset.
(0.04 MB DOC)

Table S10 Prior distributions and means of mutation rates and effective population sizes used for all coalescent simulations.
(0.03 MB DOC)

Text S1 Rationale of the study and supplementary analyses.
(0.08 MB DOC)
