# Supplementary figures and images for: Inferring the Demographic History of African Farmers and Pygmy Hunter–Gatherers Using a Multilocus Resequencing Data Set
Source: PLoS Genet. 2009 Apr 10;5(4):e1000448. doi: 10.1371/journal.pgen.1000448 (PMC2661362; doi:10.1371/journal.pgen.1000448)

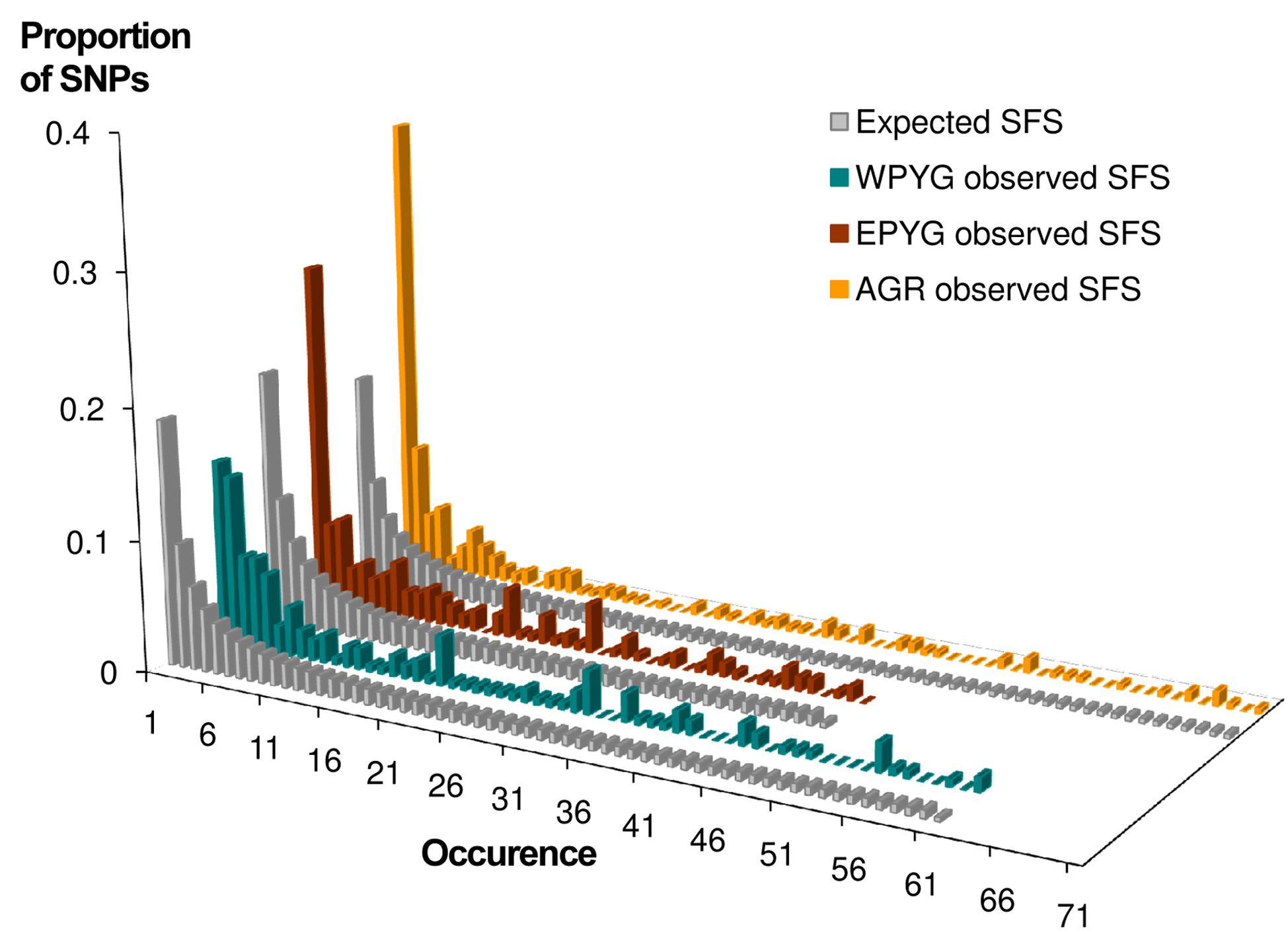

Supplement: Figure S1 — Site frequency spectra of the WPYG, EPYG and AGR populations for the 20 autosomal regions, using the composite population dataset. Gray histograms represent the expected SFS of a constant-sized panmictic population with the same number of individuals as observed in the three population groups. The right tail of the agricultural SFS has been truncated for clarity. (8.29 MB TIF) [file pgen.1000448.s001.tif]

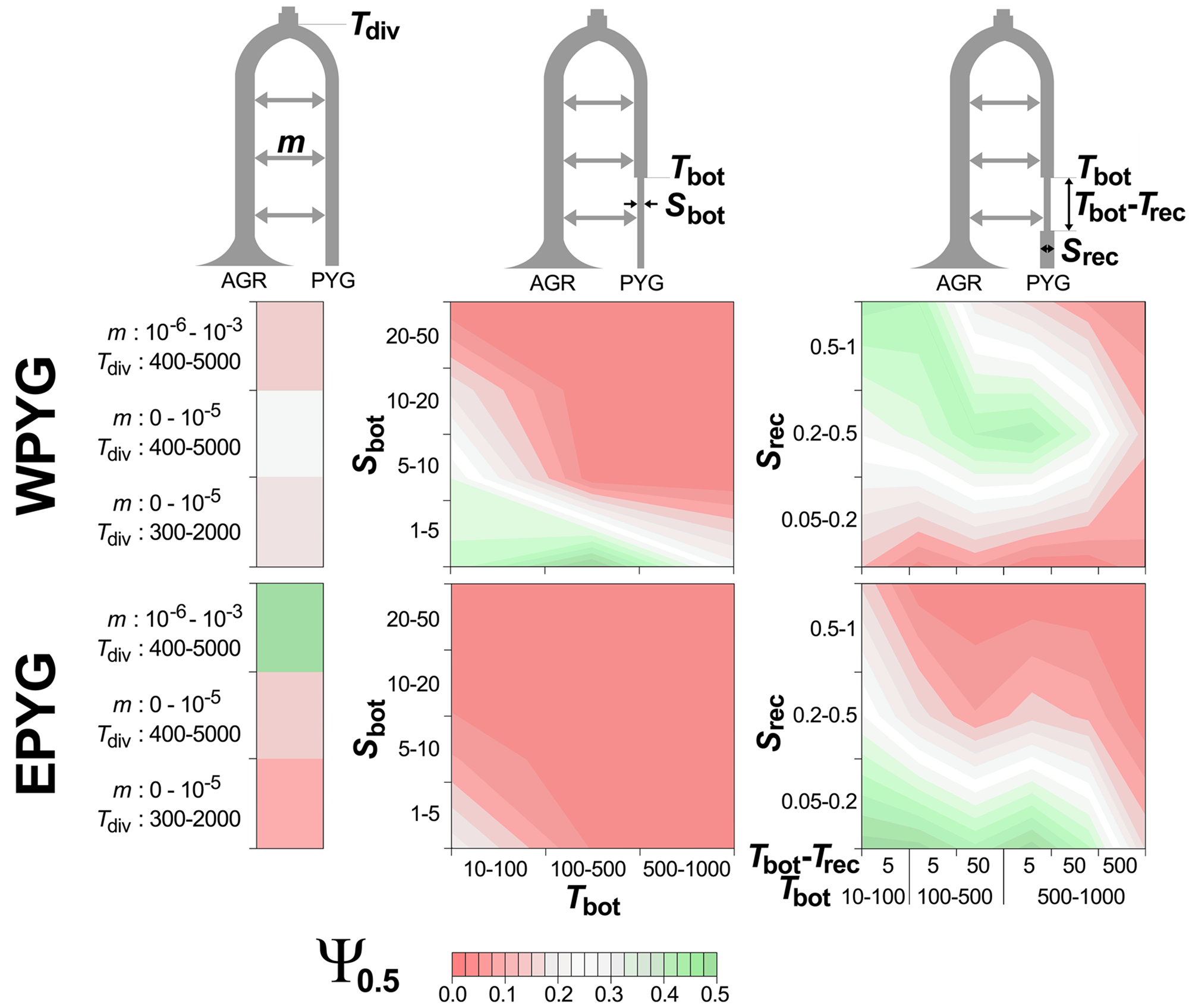

Supplement: Figure S2 — Different models simulating the demographic regime of the WPYG and EPYG groups and the mean proportion of small distances (Ψ 0.5) obtained in comparisons with simulated statistics, based on the composite population dataset. Times are in generations. T bot and S bot are the time and strength of the bottleneck, respectively. T rec and S rec are the time and strength of the population size recovery, respectively. Modeling details and the prior distributions of parameters are given in Table S6. We calculated the mean Ψ 0.5 for a given model and set of parameters, by resampling, among 100,000 simulations, 100 sets of 10,000 simulations of the model, calculating Ψ 0.5 for each set and reporting the mean Ψ 0.5 across sets. The model with one bottleneck (T bot: 10–100 generations, S bot = 5) and one recovery (T rec = T bot - 5 generations, S rec: 0.5–1) generated, for WPYG, the maximum Ψ 0.5 in 62% of cases when compared with all models and in 98% of cases when compared with only constant population size models. For the EPYG group, the constant population size model generated the maximum Ψ 0.5 in 56% of cases when compared with all models. (10.20 MB TIF) [file pgen.1000448.s002.tif]

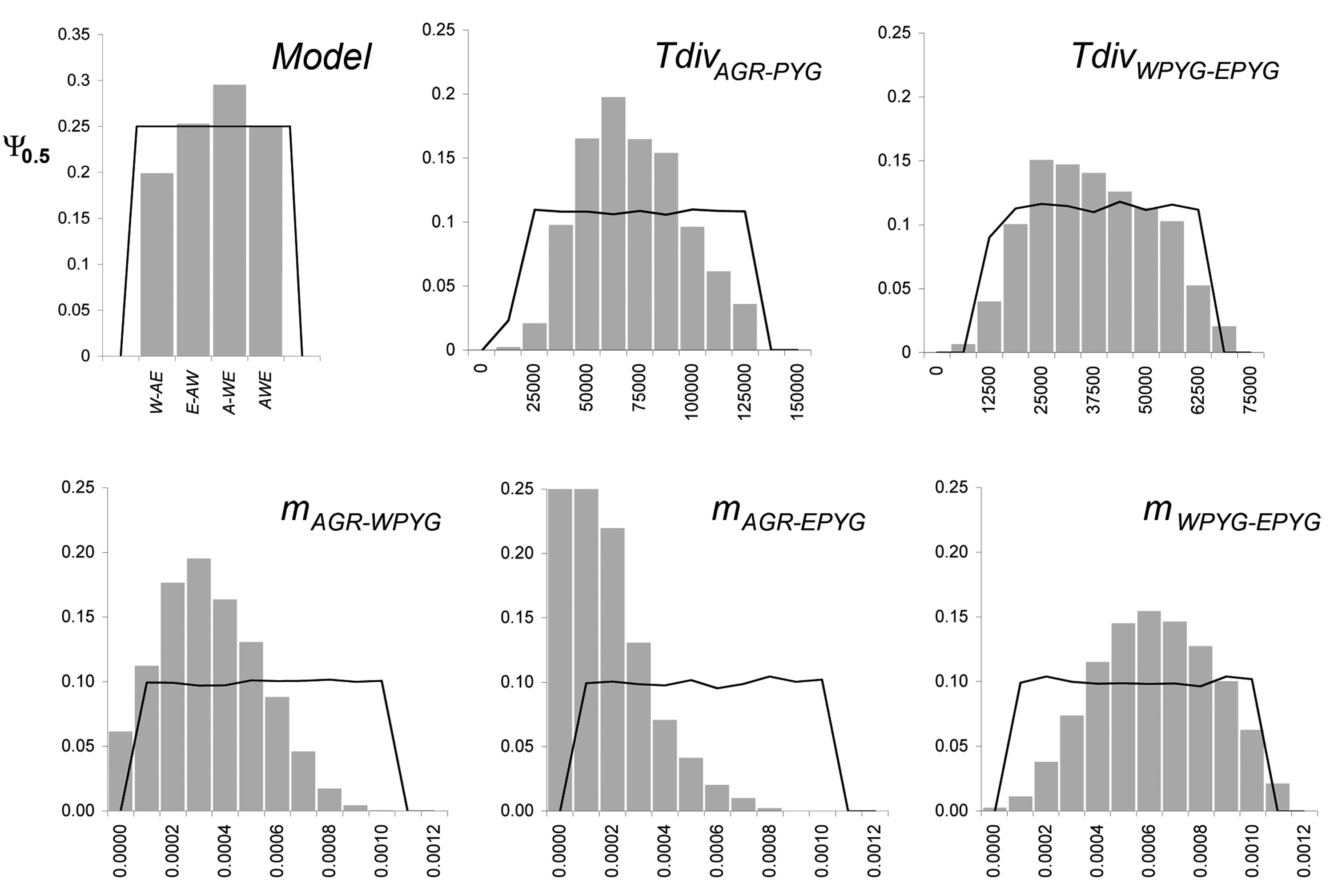

Supplement: Figure S3 — Prior and approximated posterior distributions of the IM model and IM parameters under the best-fit A-WE model for the composite population dataset. Divergence times Tdiv are expressed in years and migration rates m in proportion of migrants per generation. Black lines represent prior distributions and gray histograms represent approximated posterior distributions obtained by the ABC method [37], except for model choice, for which the posterior distribution was estimated based on the proportions of small distances generated by each model (Materials and Methods). We observed a highly significant negative correlation between ξ - the threshold at which distances between simulated and observed statistics are considered to be “small” (Materials and Methods) - and the proportion of small distances Ψξ generated by the A-WE model (r 2 = 0.946, P<0.0001). The joint approximated posterior distribution of Tdiv WPYG-EPYG and m WPYG-EPYG is shown in Figure S4. (6.07 MB TIF) [file pgen.1000448.s003.tif]

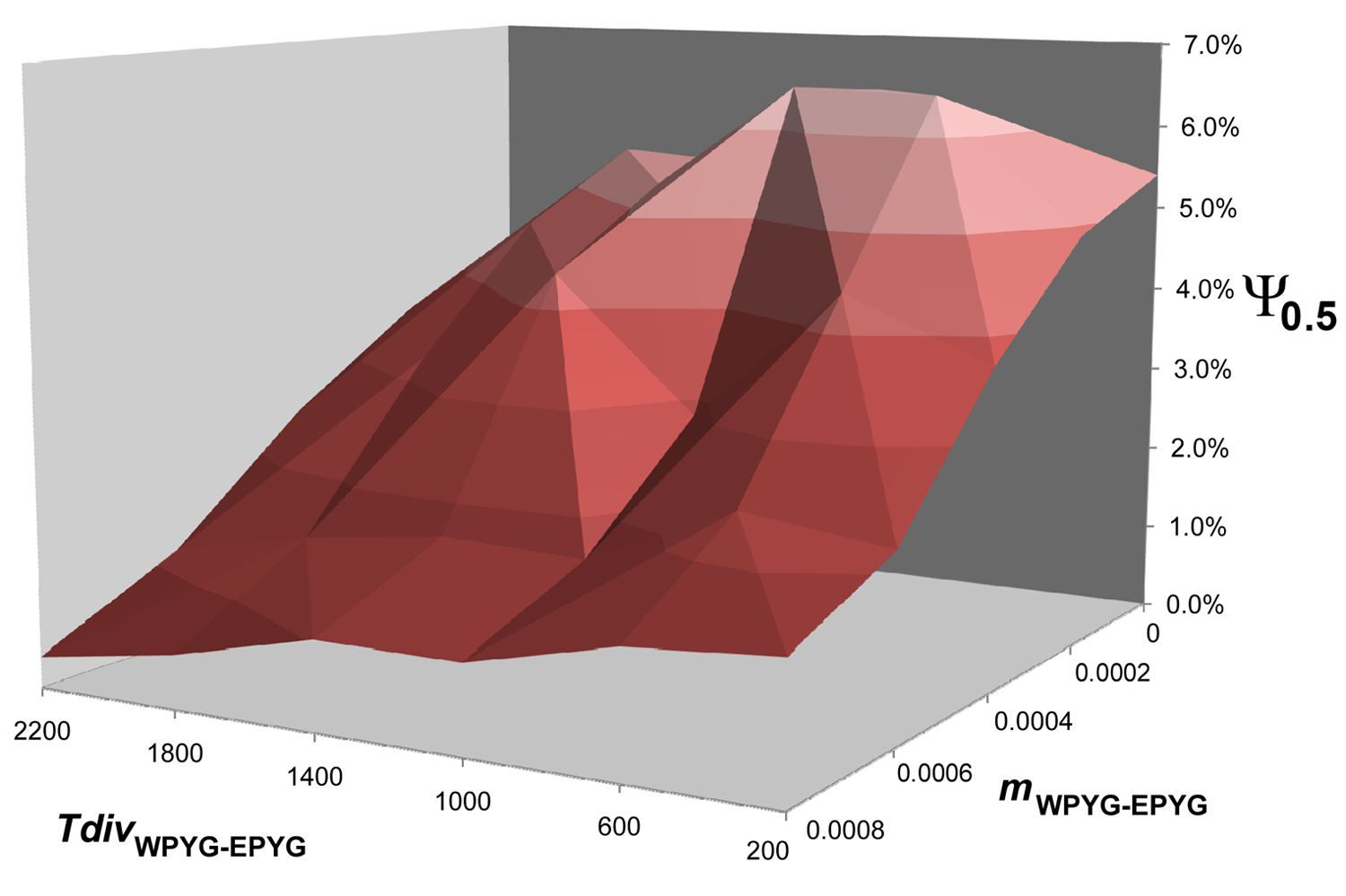

Supplement: Figure S4 — Approximated joint posterior distribution of the time of divergence and migration rate between Western and Eastern Pygmies for the composite population dataset. The posterior distribution of the two parameters is estimated by means of the proportion of small distances Ψ 0.5. The time of divergence Tdiv WPYG-EPYG and the migration rate m WPYG-EPYG are reported in generations and in proportion of migrants per generation, respectively. (4.52 MB TIF) [file pgen.1000448.s004.tif]

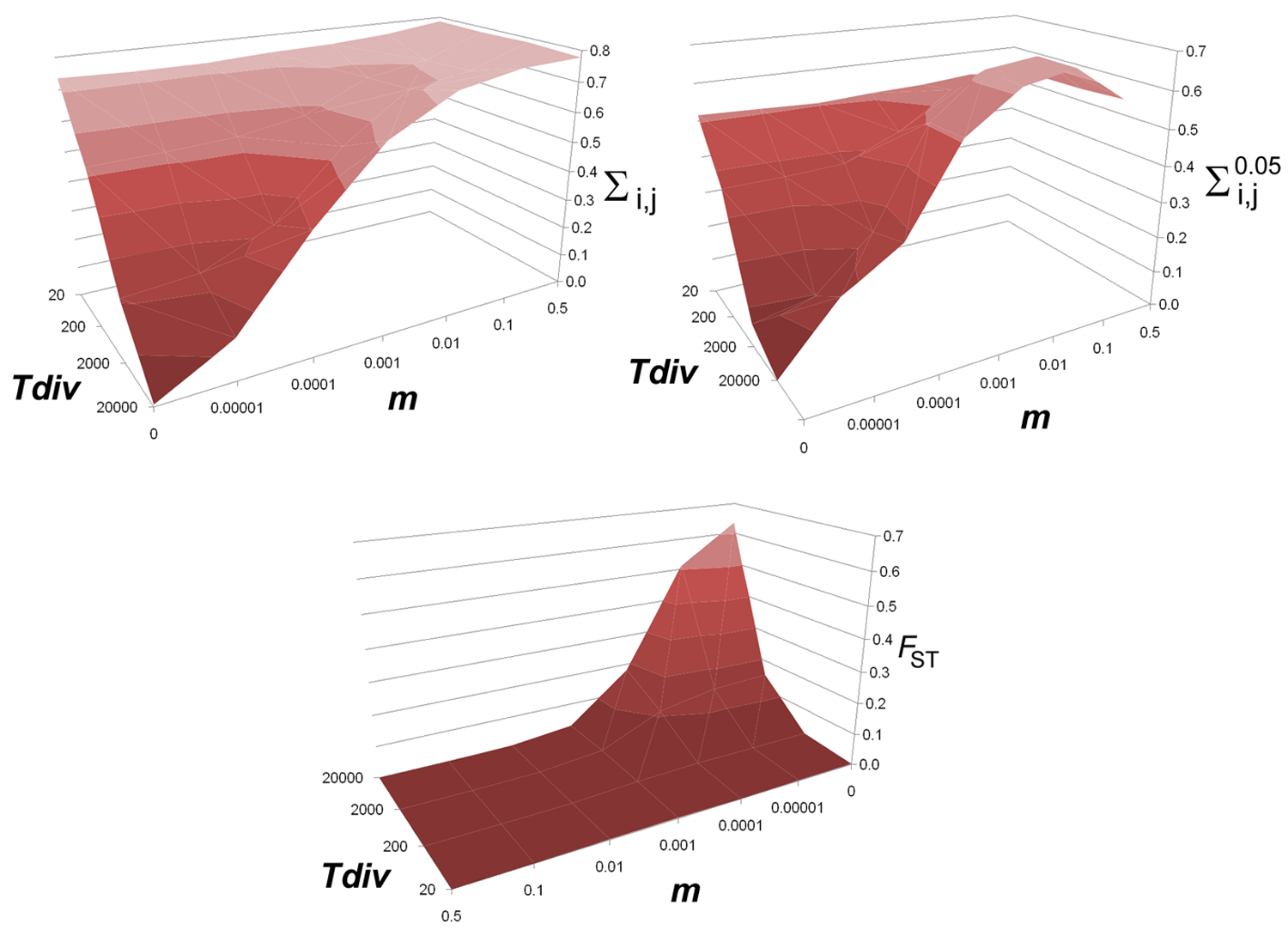

Supplement: Figure S5 — Behavior of selected summary statistics under various levels of divergence and gene flow. Time of divergence (in generations) and migration rate (in proportion of migrants per generation) are represented by Tdiv and m, respectively. (4.93 MB TIF) [file pgen.1000448.s005.tif]
