# Supplementary material for: Inferring the Demographic History of African Farmers and Pygmy Hunter–Gatherers Using a Multilocus Resequencing Data Set
Source: PLoS Genet. 2009 Apr 10;5(4):e1000448. doi: 10.1371/journal.pgen.1000448 (PMC2661362; doi:10.1371/journal.pgen.1000448)
Supplement: Table S1 — Location of the 25 resequenced regions and their respective distances to coding regions. (0.09 MB DOC) [file pgen.1000448.s006.doc]

| **Region** | **Chr** | **Length (bp)** | **Position** | **5' closest EST** | **Distance (bp)** | **3' closest EST** | **Distance (bp)** | **5' closest gene** | **Distance (bp)** | **3' closest gene** | **Distance (bp)** |
| --- | --- | --- | --- | --- | --- | --- | --- | --- | --- | --- | --- |
| R04 | 1p | 1328 | 106787624-106788951 | *AA954094* | 358983 | *BU854483* | 373433 | *BC043293* | 824544 | *PRMT6* | 611981 |
| R05 | 2p | 1271 | 76163330-76164600 | *BF967228* | 363160 | *DB302076* | 664592 | *c2orf3* | 371500 | *UNQ3075* | 664592 |
| R06 | 2q | 1300 | 117090864-117092163 | *BC030832* | 772458 | *DA742384* | 771905 | *DPP10* | 772458 | *DQ571524* | 405462 |
| R07 | 3p | 1373 | 20665087-20666459 | *AK026452* | 258029 | *CF618845* | 255538 | *SGOL1* | 462400 | *HPX-42* | 755763 |
| R08 | 3q | 1371 | 146671295-146672665 | *AI693058* | 460613 | *CA453629* | 348970 | *C3orf58* | 1477404 | *DQ595575* | 352302 |
| R09 | 4p | 1256 | 29547438-29548693 | *DB451145* | 269619 | *BG216723* | 727133 | *FLJ45721* | 2718314 | *PCDH7* | 782442 |
| R10 | 4q | 1326 | 179693433-179694758 | *BC033326* | 544535 | *BC043428* | 438222 | *BC033326* | 544535 | *AF088005* | 2527479 |
| R14 | 6q | 1300 | 91757485-91758784 | *BE044076* | 400143 | *BC037927* | 636691 | *MAP3K7* | 403857 | *BC037927* | 636691 |
| R16 | 7p | 1401 | 13052349-13053749 | *BU664973* | 259878 | *DA181168* | 53792 | *ARL4A* | 355266 | *ETV1* | 843632 |
| R17 | 7q | 1350 | 118479068-118480417 | *AA018891* | 564369 | *CB338058* | 566302 | *ANKRD7* | 809087 | *KCND2* | 1220541 |
| R18 | 8p | 1256 | 5139032-5140287 | *DB446481* | 167686 | *BC040995* | 210712 | *CSMD1* | 299296 | *CR623475* | 1108198 |
| R20 | 8q | 1282 | 137174739-137176020 | *DB092925* | 405774 | *DA593750* | 435281 | *KHDRBS3* | 445709 | *C8ORFK32* | 2035428 |
| R21 | 9p | 1390 | 11567765-11569154 | *DA212144* | 223691 | *DB098556* | 39340 | *PTPRD* | 3263519 | *TYRP1* | 1131103 |
| R25 | 11q | 1340 | 80750645-80751984 | *AK001959* | 525369 | *BC041900* | 516560 | *AF009227* | 2146040 | *BC041900* | 516560 |
| R26 | 11q | 1330 | 96959343-96960672 | *BF575990* | 292539 | *BG182718* | 358300 | *JRKL* | 1192968 | *CNTN5* | 1436409 |
| R30 | 14q | 1345 | 82997591-82998935 | *DB451719* | 418416 | *AI217957* | 444034 | *BC029835* | 1838433 | *BX248253* | 2062295 |
| R32 | 15q | 1301 | 84958820-84960120 | *CD359326* | 297622 | *AK096897* | 300270 | *AGBL1* | 318370 | *TMEM83* | 961044 |
| R33 | 16q | 1350 | 58084866-58086215 | *AK057513* | 384487 | *AA782991* | 215183 | *GOT2* | 759119 | *CDH8* | 2158521 |
| R36 | 18q | 1200 | 26002122-26003321 | *BG461864* | 619942 | *DA340389* | 543921 | *CDH2* | 1990933 | *CR600534* | 820651 |
| R42 | 20q | 1347 | 53069630-53070976 | *BC008992* | 368795 | *DB033847* | 271799 | *DOK5* | 368513 | *CBLN4* | 934927 |
| R38 | Xq | 1350 | 93991910-93993259 | *BG482705* | 249987 | *BX509718* | 563633 | *RP1-32F7.2* | 1137993 | *DIAPH2* | 1833106 |
| R44 | Xp | 1370 | 5101635-5103004 | *DA565271* | 478669 | *R05989* | 478458 | *BC017239* | 1252887 | *NLGN4X* | 715079 |
| R41 | Y | 1311 | 9036-10235 | - | - | - | - | *OK/SW-cl.16* | - | *-* | - |
| R43 | Yq | 1100 | 6799252-6800562 | - | - | - | - | *AMELY* | - | *-* | - |
| R40 | mtDNA | 1200 | 13434587-13435686 | - | - | - | - | *USP9Y* | - | *-* | - |
| **Total** |  | 32748 |  |  |  |  |  |  |  |  |  |
| **Mean** |  | 1310 |  |  | 394762 |  | 418821 |  | 1079688 |  | 1114282 |
| **Minimum** |  | 1100 |  |  | 167686 |  | 39340 |  | 299296 |  | 352302 |
| **Maximum** |  | 1401 |  |  | 772458 |  | 771905 |  | 3263519 |  | 2527479 |

**Table S1.** Location of the 25 resequenced regions and their respective distances to coding regions

ESTs refer here to mRNAs or spliced ESTs and genes refer to UCSC gene predictions or RefSeq genes. Regions located in the non-recombining portion of the Y-chromosome and mtDNA were selected within genes, as reported in a previous study [57], since hitchhiking effects with adjacent genes can not be avoided in the absence of recombination.
