# Supplementary material for: Inferring the Demographic History of African Farmers and Pygmy Hunter–Gatherers Using a Multilocus Resequencing Data Set
Source: PLoS Genet. 2009 Apr 10;5(4):e1000448. doi: 10.1371/journal.pgen.1000448 (PMC2661362; doi:10.1371/journal.pgen.1000448)
Supplement: Table S2 — Mean pairwise F ST values among the 12 sub-Saharan African populations for (A) 20 autosomal regions, (B) two X regions, (C) one Y region and (D) one mtDNA region. (0.14 MB DOC) [file pgen.1000448.s007.doc]

**Table S2.** Mean pairwise *F*_ST_ values among the 12 sub-Saharan African populations for (A) 20 autosomal regions, (B) two X regions, (C) one Y region and (D) one mtDNA region

| **A** | Biaka | C. Baka | G. Baka | Bakola | Mbuti | N. Twa | S. Twa | Yoruba | Akele | Ngumba | Chagga | Mozambicans |
| --- | --- | --- | --- | --- | --- | --- | --- | --- | --- | --- | --- | --- |
| Biaka |  | - | - | + | + | + | + | + | + | + | + | + |
| C. Baka | 0.010 |  | - | + | + | + | + | + | + | + | + | + |
| G. Baka | 0.012 | 0.002 |  | + | + | - | + | + | + | + | + | + |
| Bakola | 0.019 | 0.047 | 0.035 |  | + | + | + | + | + | + | + | + |
| Mbuti | 0.035 | 0.053 | 0.045 | 0.046 |  | + | + | + | + | + | + | + |
| N. Twa | 0.034 | 0.041 | 0.009 | 0.045 | 0.031 |  | - | + | + | + | + | + |
| S. Twa | 0.031 | 0.050 | 0.022 | 0.025 | 0.041 | 0.022 |  | + | + | + | + | + |
| Yoruba | 0.033 | 0.035 | 0.034 | 0.027 | 0.051 | 0.033 | 0.022 |  | - | - | - | - |
| Akele | 0.024 | 0.033 | 0.022 | 0.021 | 0.047 | 0.021 | 0.023 | -0.001 |  | - | - | - |
| Ngumba | 0.026 | 0.038 | 0.032 | 0.021 | 0.051 | 0.029 | 0.016 | -0.007 | -0.008 |  | - | - |
| Chagga | 0.035 | 0.042 | 0.040 | 0.033 | 0.054 | 0.036 | 0.042 | 0.002 | 0.001 | -0.004 |  | - |
| Mozambicans | 0.046 | 0.057 | 0.047 | 0.038 | 0.061 | 0.032 | 0.031 | 0.015 | 0.009 | 0.009 | 0.010 |  |

| **B** | Biaka | C. Baka | G. Baka | Bakola | Mbuti | N. Twa | S. Twa | Yoruba | Akele | Ngumba | Chagga | Mozambicans |
| --- | --- | --- | --- | --- | --- | --- | --- | --- | --- | --- | --- | --- |
| Biaka |  | - | - | + | - | - | - | - | - | - | - | - |
| C. Baka | 0.050 |  | - | - | - | - | - | - | - | - | - | - |
| G. Baka | 0.108 | 0.046 |  | - | - | - | - | - | - | - | - | - |
| Bakola | 0.140 | -0.001 | 0.052 |  | - | - | - | - | - | - | - | - |
| Mbuti | -0.003 | 0.059 | 0.084 | 0.171 |  | + | + | - | - | + | - | - |
| N. Twa | 0.098 | -0.039 | 0.045 | -0.042 | 0.120 |  | - | - | - | - | - | - |
| S. Twa | 0.107 | -0.018 | 0.006 | -0.066 | 0.125 | -0.045 |  | - | - | - | - | - |
| Yoruba | 0.079 | -0.007 | -0.009 | 0.024 | 0.064 | 0.005 | -0.013 |  | - | - | - | - |
| Akele | 0.067 | -0.049 | 0.037 | -0.064 | 0.085 | -0.057 | -0.066 | -0.016 |  | - | - | - |
| Ngumba | 0.104 | -0.023 | 0.062 | -0.030 | 0.107 | -0.033 | -0.043 | 0.003 | -0.066 |  | - | - |
| Chagga | 0.062 | 0.011 | -0.026 | 0.032 | 0.053 | 0.021 | -0.004 | -0.013 | 0.002 | 0.027 |  | - |
| Mozambicans | 0.078 | -0.030 | 0.002 | -0.005 | 0.066 | -0.019 | -0.032 | -0.030 | -0.038 | -0.022 | -0.014 |  |

| **C** | Biaka | C. Baka | G. Baka | Bakola | Mbuti | N. Twa | Yoruba | Akele | Ngumba | Chagga | Mozambicans |
| --- | --- | --- | --- | --- | --- | --- | --- | --- | --- | --- | --- |
| Biaka |  | - | - | - | - | - | - | - | - | - | - |
| C. Baka | -0.064 |  | - | + | - | - | + | + | + | + | - |
| G. Baka | 0.005 | -0.048 |  | - | - | - | + | + | + | + | - |
| Bakola | -0.053 | 0.105 | 0.097 |  | - | - | - | + | - | - | - |
| Mbuti | -0.135 | -0.034 | -0.042 | -0.008 |  | - | - | + | - | + | - |
| N. Twa | -0.231 | 0.010 | 0.066 | 0.013 | -0.059 |  | - | - | - | - | - |
| Yoruba | 0.088 | 0.266 | 0.290 | 0.044 | 0.139 | 0.103 |  | - | - | - | - |
| Akele | 0.336 | 0.367 | 0.431 | 0.166 | 0.325 | 0.242 | 0.039 |  | - | - | - |
| Ngumba | 0.057 | 0.239 | 0.259 | 0.026 | 0.109 | 0.080 | -0.062 | 0.055 |  | - | - |
| Chagga | 0.055 | 0.293 | 0.329 | 0.086 | 0.177 | 0.087 | -0.019 | -0.019 | -0.017 |  | - |
| Mozambicans | -0.167 | 0.044 | 0.070 | 0.006 | -0.041 | -0.086 | 0.050 | 0.125 | 0.034 | 0.058 |  |

| **D** | Biaka | C_Baka | G_Baka | Bakola | Mbuti | N. Twa | S. Twa | Yoruba | Akele | Ngumba | Chagga | Mozambicans |
| --- | --- | --- | --- | --- | --- | --- | --- | --- | --- | --- | --- | --- |
| Biaka |  | + | - | - | + | + | + | + | + | - | + | + |
| C. Baka | 0.099 |  | - | - | + | + | + | + | + | + | + | + |
| G. Baka | 0.047 | -0.025 |  | - | + | + | + | + | + | + | + | + |
| Bakola | 0.025 | 0.097 | 0.003 |  | + | + | + | + | + | + | + | + |
| Mbuti | 0.256 | 0.429 | 0.405 | 0.372 |  | - | - | + | + | + | + | + |
| N. Twa | 0.295 | 0.484 | 0.466 | 0.436 | 0.009 |  | - | + | + | + | + | + |
| S. Twa | 0.381 | 0.589 | 0.575 | 0.558 | 0.080 | 0.005 |  | + | + | + | + | + |
| Yoruba | 0.080 | 0.126 | 0.074 | 0.056 | 0.311 | 0.368 | 0.450 |  | + | + | + | + |
| Akele | 0.032 | 0.152 | 0.101 | 0.056 | 0.160 | 0.187 | 0.260 | 0.041 |  | + | + | + |
| Ngumba | 0.021 | 0.273 | 0.228 | 0.169 | 0.193 | 0.234 | 0.351 | 0.146 | 0.048 |  | - | + |
| Chagga | 0.086 | 0.340 | 0.299 | 0.251 | 0.127 | 0.163 | 0.289 | 0.208 | 0.070 | -0.022 |  | + |
| Mozambicans | 0.240 | 0.431 | 0.409 | 0.375 | 0.104 | 0.135 | 0.271 | 0.328 | 0.163 | 0.140 | 0.083 |  |

For each Table, the upper matrix reports statistical significance (the plus symbol represents significant *F*_ST_, the minus symbol represent non-significant *F*_ST_). Only related samples were excluded from this analysis.
