# Supplementary material for: Inferring the Demographic History of African Farmers and Pygmy Hunter–Gatherers Using a Multilocus Resequencing Data Set
Source: PLoS Genet. 2009 Apr 10;5(4):e1000448. doi: 10.1371/journal.pgen.1000448 (PMC2661362; doi:10.1371/journal.pgen.1000448)
Supplement: Table S3 — Mean diversity indices and neutrality tests across the 24 independent genomic regions sequenced in the composite population dataset of WPYG, EPYG and AGR. (0.05 MB DOC) [file pgen.1000448.s008.doc]

**Table S3.** Mean diversity indices and neutrality tests across the 24 independent genomic regions sequenced in the composite population dataset of WPYG, EPYG and AGR.

|  |  | *S* | *π* | *θ*_W_ | T*D*^a,b^ | *D**^a,b^ | *Fs*^a,b^ |
| --- | --- | --- | --- | --- | --- | --- | --- |
| *20 autosomal regions* | |  |  |  |  |  |  |
|  | WPYG | 183 | 0.00123 | 0.00129 | -0.100 | 0.334 | -0.995 |
|  | EPYG | 186 | 0.00130 | 0.00138 | -0.141 | -0.421 | -1.593 |
|  | AGR | 270 | 0.00116 | 0.00172 | **-0.785** | **-1.686** | **-4.700** |
|  |  |  |  |  |  |  |  |
| *Two X-linked regions* | |  |  |  |  |  |  |
|  | WPYG | 7 | 0.00085 | 0.00052 | 1.221 | 0.905 | 0.623 |
|  | EPYG | 9 | 0.00085 | 0.00068 | 0.626 | -0.013 | -0.255 |
|  | AGR | 13 | 0.00089 | 0.00087 | 0.194 | -1.234 | -0.988 |
|  |  |  |  |  |  |  |  |
| *One Y-linked region* | |  |  |  |  |  |  |
|  | WPYG | 6 | 0.00040 | 0.00057 | -0.763 | -0.503 | -2.230 |
|  | EPYG | 2 | 0.00032 | 0.00024 | 0.769 | 0.885 | 0.536 |
|  | AGR | 6 | 0.00014 | 0.00050 | **-1.656** | **-2.595** | **-4.084** |
|  |  |  |  |  |  |  |  |
| *One mtDNA region* | |  |  |  |  |  |  |
|  | WPYG | 18 | 0.00175 | 0.00321 | -1.384 | -2.160 | -7.056 |
|  | EPYG | 20 | 0.00327 | 0.00377 | -0.427 | 0.228 | -8.871 |
|  | AGR | 42 | 0.0031 | 0.00666 | -1.661 | -2.081 | **-69.680** |

^a^T*D*: Tajima’s *D*; *D**: Fu & Li’s *D**; *Fs*: Fu’s *Fs*

^b^Neutrality statistics in bold are statistically significant at the 5% level for all tests, except Fu’s *Fs* (set at 2%). Variances of neutrality statistics across autosomal regions are reported in Table S4.
