# Supplementary material for: Inferring the Demographic History of African Farmers and Pygmy Hunter–Gatherers Using a Multilocus Resequencing Data Set
Source: PLoS Genet. 2009 Apr 10;5(4):e1000448. doi: 10.1371/journal.pgen.1000448 (PMC2661362; doi:10.1371/journal.pgen.1000448)
Supplement: Table S4 — Variances of statistics from sequence-based neutrality tests across the 20 autosomal regions in WPYG, EPYG and AGR populations, using the filtered and composite population datasets. (0.03 MB DOC) [file pgen.1000448.s009.doc]

**Table S4.** Variances of statistics from sequence-based neutrality tests across the 20 autosomal regions in WPYG, EPYG and AGR populations, using the filtered and composite population datasets

|  | V(T*D*^a^) | V(*D**^a^) | V(*Fs*^a^) |
| --- | --- | --- | --- |
|  | |  |  |
| Filtered population Dataset | |  |  |
| WPYG | 0.394 | 0.594 | 3.176 |
| EPYG | 0.836 | 0.533 | 3.256 |
| AGR | **0.330**^b^ | 0.941 | 3.385 |
|  | |  |  |
| Composite population Dataset | |  |  |
| WPYG | 0.611 | 0.831 | 5.630 |
| EPYG | 0.640 | 1.219 | 7.732 |
| AGR | **0.373**^b^ | 1.1744 | 11.489 |

^a^T*D*: Tajima’s *D*; *D**: Fu & Li’s *D**; *Fs*: Fu’s *Fs*

^b^Values in bold are statistically significant at the 5% level
