# Supplementary material for: Inferring the Demographic History of African Farmers and Pygmy Hunter–Gatherers Using a Multilocus Resequencing Data Set
Source: PLoS Genet. 2009 Apr 10;5(4):e1000448. doi: 10.1371/journal.pgen.1000448 (PMC2661362; doi:10.1371/journal.pgen.1000448)
Supplement: Table S5 — Mean summary statistics for genetic differentiation between the WPYG, EPYG and AGR populations, across the 24 genomic regions, for the filtered and composite population datasets. (0.06 MB DOC) [file pgen.1000448.s010.doc]

**Table S5.** Mean summary statistics for genetic differentiation between the WPYG, EPYG and AGR populations, across the 24 genomic regions, for the filtered and composite population datasets

|  | | *F*_ST_ | $\Sigma_{i,j}$(%)^a^ | $\Sigma_{i,j}^{0.05}$(%)^a^ | $\hat{p}_{sS}$^a^ |
| --- | --- | --- | --- | --- | --- |
|  | | |  |  |  |
| **Filtered population dataset** | | |  |  |  |
| *20 autosomal regions* | | |  |  |  |
| WPYG/EPYG | | 0.042 | 53.8 | 13.2 | 0.200 |
| WPYG/AGR | | 0.043 | 57.1 | 24.2 | 0.163 |
| EPYG/AGR | | 0.057 | 47.8 | 13.5 | 0.185 |
| *Two X-linked regions* | | |  |  |  |
| WPYG/EPYG | | 0.003 | 75.0 | 0.0 | 0.236 |
| WPYG/AGR | | 0.026 | 77.5 | 16.7 | 0.222 |
| EPYG/AGR | | 0.049 | 60.0 | 0.0 | 0.223 |
| *One Y-linked region* | | |  |  |  |
| WPYG/EPYG | | 0.046 | 50 | 0 | 0.329 |
| WPYG/AGR | | 0.333 | 25 | 0 | 0.256 |
| EPYG/AGR | | 0.067 | 50 | 0 | 0.121 |
| *One mtDNA region* | | |  |  |  |
| WPYG/EPYG | | 0.378 | 30.4 | 28.6 | 0.162 |
| WPYG/AGR | | 0.060 | 39.5 | 60.0 | 0.086 |
| EPYG/AGR | | 0.219 | 37.8 | 28.6 | 0.131 |
|  | | |  |  |  |
| **Composite population dataset** | | |  |  |  |
| *20 autosomal regions* | | |  |  |  |
| WPYG/EPYG | 0.022 | | 55.4 | 25.8 | 0.160 |
| WPYG/AGR | 0.026 | | 51.3 | 38.5 | 0.132 |
| EPYG/AGR | 0.032 | | 45.7 | 40.4 | 0.137 |
| *Two X-linked regions* | | |  |  |  |
| WPYG/EPYG | -0.010 | | 80.0 | 16.7 | 0.235 |
| WPYG/AGR | 0.007 | | 59.7 | 16.7 | 0.233 |
| EPYG/AGR | 0.003 | | 47.2 | 16.7 | 0.231 |
| *One Y-linked region* | | |  |  |  |
| WPYG/EPYG | -0.014 | | 33.3 | 0 | 0.270 |
| WPYG/AGR | 0.184 | | 20.0 | 0 | 0.141 |
| EPYG/AGR | 0.116 | | 33.3 | 0 | 0.099 |
| *One mtDNA region* | | |  |  |  |
| WPYG/EPYG | 0.394 | | 22.6 | 28.6 | 0.172 |
| WPYG/AGR | 0.102 | | 36.4 | 50.0 | 0.088 |
| EPYG/AGR | 0.170 | | 34.8 | 37.5 | 0.126 |

^a^ $\Sigma_{i,j}$: proportion of shared mutations between pairs of populations; $\Sigma_{i,j}^{0.05}$: proportion of low-frequency shared mutations between pairs of populations; $\hat{p}_{sS}$: mean frequency of shared mutations between pairs of populations
