# Supplementary material for: Inferring the Demographic History of African Farmers and Pygmy Hunter–Gatherers Using a Multilocus Resequencing Data Set
Source: PLoS Genet. 2009 Apr 10;5(4):e1000448. doi: 10.1371/journal.pgen.1000448 (PMC2661362; doi:10.1371/journal.pgen.1000448)
Supplement: Table S6 — Prior distributions of the parameters of 33 models simulated to assess the demographic regime of Pygmy population groups, using the composite population dataset. (0.12 MB DOC) [file pgen.1000448.s011.doc]

**Table S6.** Prior distributions of the parameters of 33 models simulated to assess the demographic regime of Pygmy population groups, using the composite population dataset

|  | PYG-AGR divergence parameters | |  | AGR expansion parameters | | | |  | PYG bottleneck and recovery parameters | | | |
| --- | --- | --- | --- | --- | --- | --- | --- | --- | --- | --- | --- | --- |
|  | *Tdiv* | *m* |  | *Texp_1_* | *Texp_2_* | *r_1_* | *r_2_* |  | *Tbot_1_* | *Sbot_1_* | *Trec_1_* | *Srec_1_* |
|  |  |  |  |  |  |  |  |  |  |  |  |  |
| Constant | [400-5000] | [10^-6^-10^-3^] |  | [2000-2500] | [200-300] | [0.4-0.6] | [0.02-0.04] |  |  |  |  |  |
| Constant_lowmig | [400-5000] | [0-10^-5^] |  | [2000-2500] | [200-300] | [0.4-0.6] | [0.02-0.04] |  |  |  |  |  |
| Constant_lowdivmig | [300-2000] | [0-10^-5^] |  | [2000-2500] | [200-300] | [0.4-0.6] | [0.02-0.04] |  |  |  |  |  |
|  |  |  |  |  |  |  |  |  |  |  |  |  |
| 1Bottleneck_T_10_100_N_1_5 | [400-5000] | [10^-6^-10^-3^] |  | [2000-2500] | [200-300] | [0.4-0.6] | [0.02-0.04] |  | [10-100] | [1-5] |  |  |
| 1Bottleneck_T_10_100_N_5_10 | [400-5000] | [10^-6^-10^-3^] |  | [2000-2500] | [200-300] | [0.4-0.6] | [0.02-0.04] |  | [10-100] | [5-10] |  |  |
| 1Bottleneck_T_10_100_N_10_20 | [400-5000] | [10^-6^-10^-3^] |  | [2000-2500] | [200-300] | [0.4-0.6] | [0.02-0.04] |  | [10-100] | [10-20] |  |  |
| 1Bottleneck_T_10_100_N_20_50 | [400-5000] | [10^-6^-10^-3^] |  | [2000-2500] | [200-300] | [0.4-0.6] | [0.02-0.04] |  | [10-100] | [20-50] |  |  |
| 1Bottleneck_T_100_500_N_1_5 | [400-5000] | [10^-6^-10^-3^] |  | [2000-2500] | [200-300] | [0.4-0.6] | [0.02-0.04] |  | [100-500] | [1-5] |  |  |
| 1Bottleneck_T_100_500_N_5_10 | [400-5000] | [10^-6^-10^-3^] |  | [2000-2500] | [200-300] | [0.4-0.6] | [0.02-0.04] |  | [100-500] | [5-10] |  |  |
| 1Bottleneck_T_100_500_N_10_20 | [400-5000] | [10^-6^-10^-3^] |  | [2000-2500] | [200-300] | [0.4-0.6] | [0.02-0.04] |  | [100-500] | [10-20] |  |  |
| 1Bottleneck_T_100_500_N_20_50 | [400-5000] | [10^-6^-10^-3^] |  | [2000-2500] | [200-300] | [0.4-0.6] | [0.02-0.04] |  | [100-500] | [20-50] |  |  |
| 1Bottleneck_T_500_1000_N_1_5 | [400-5000] | [10^-6^-10^-3^] |  | [2000-2500] | [200-300] | [0.4-0.6] | [0.02-0.04] |  | [500-1000] | [1-5] |  |  |
| 1Bottleneck_T_500_1000_N_5_10 | [400-5000] | [10^-6^-10^-3^] |  | [2000-2500] | [200-300] | [0.4-0.6] | [0.02-0.04] |  | [500-1000] | [5-10] |  |  |
| 1Bottleneck_T_500_1000_N_10_20 | [400-5000] | [10^-6^-10^-3^] |  | [2000-2500] | [200-300] | [0.4-0.6] | [0.02-0.04] |  | [500-1000] | [10-20] |  |  |
| 1Bottleneck_T_500_1000_N_20_50 | [400-5000] | [10^-6^-10^-3^] |  | [2000-2500] | [200-300] | [0.4-0.6] | [0.02-0.04] |  | [500-1000] | [20-50] |  |  |
|  |  |  |  |  |  |  |  |  |  |  |  |  |
| 1Bott_1Recov_T_10_t_5_s_0.05 | [400-5000] | [10^-6^-10^-3^] |  | [2000-2500] | [200-300] | [0.4-0.6] | [0.02-0.04] |  | [10-100] | 5 | Tbot_1_ - 5 | 0.05 |
| 1Bott_1Recov_T_10_t_5_s_0.2 | [400-5000] | [10^-6^-10^-3^] |  | [2000-2500] | [200-300] | [0.4-0.6] | [0.02-0.04] |  | [10-100] | 5 | Tbot_1_ - 5 | 0.2 |
| 1Bott_1Recov_T_10_t_5_s_0.5 | [400-5000] | [10^-6^-10^-3^] |  | [2000-2500] | [200-300] | [0.4-0.6] | [0.02-0.04] |  | [10-100] | 5 | Tbot_1_ - 5 | 0.5 |
| 1Bott_1Recov_T_100_t_5_s_0.05 | [400-5000] | [10^-6^-10^-3^] |  | [2000-2500] | [200-300] | [0.4-0.6] | [0.02-0.04] |  | [100-500] | 5 | Tbot_1_ - 5 | 0.05 |
| 1Bott_1Recov_T_100_t_5_s_0.2 | [400-5000] | [10^-6^-10^-3^] |  | [2000-2500] | [200-300] | [0.4-0.6] | [0.02-0.04] |  | [100-500] | 5 | Tbot_1_ - 5 | 0.2 |
| 1Bott_1Recov_T_100_t_5_s_0.5 | [400-5000] | [10^-6^-10^-3^] |  | [2000-2500] | [200-300] | [0.4-0.6] | [0.02-0.04] |  | [100-500] | 5 | Tbot_1_ - 5 | 0.5 |
| 1Bott_1Recov_T_100_t_50_s_0.05 | [400-5000] | [10^-6^-10^-3^] |  | [2000-2500] | [200-300] | [0.4-0.6] | [0.02-0.04] |  | [100-500] | 5 | Tbot_1_ - 50 | 0.05 |
| 1Bott_1Recov_T_100_t_50_s_0.2 | [400-5000] | [10^-6^-10^-3^] |  | [2000-2500] | [200-300] | [0.4-0.6] | [0.02-0.04] |  | [100-500] | 5 | Tbot_1_ - 50 | 0.2 |
| 1Bott_1Recov_T_100_t_50_s_0.5 | [400-5000] | [10^-6^-10^-3^] |  | [2000-2500] | [200-300] | [0.4-0.6] | [0.02-0.04] |  | [100-500] | 5 | Tbot_1_ - 50 | 0.5 |
| 1Bott_1Recov_T_500_t_5_s_0.05 | [400-5000] | [10^-6^-10^-3^] |  | [2000-2500] | [200-300] | [0.4-0.6] | [0.02-0.04] |  | [500-1000] | 5 | Tbot_1_ - 5 | 0.05 |
| 1Bott_1Recov_T_500_t_5_s_0.2 | [400-5000] | [10^-6^-10^-3^] |  | [2000-2500] | [200-300] | [0.4-0.6] | [0.02-0.04] |  | [500-1000] | 5 | Tbot_1_ - 5 | 0.2 |
| 1Bott_1Recov_T_500_t_5_s_0.5 | [400-5000] | [10^-6^-10^-3^] |  | [2000-2500] | [200-300] | [0.4-0.6] | [0.02-0.04] |  | [500-1000] | 5 | Tbot_1_ - 5 | 0.5 |
| 1Bott_1Recov_T_500_t_50_s_0.05 | [400-5000] | [10^-6^-10^-3^] |  | [2000-2500] | [200-300] | [0.4-0.6] | [0.02-0.04] |  | [500-1000] | 5 | Tbot_1_ - 50 | 0.05 |
| 1Bott_1Recov_T_500_t_50_s_0.2 | [400-5000] | [10^-6^-10^-3^] |  | [2000-2500] | [200-300] | [0.4-0.6] | [0.02-0.04] |  | [500-1000] | 5 | Tbot_1_ - 50 | 0.2 |
| 1Bott_1Recov_T_500_t_50_s_0.5 | [400-5000] | [10^-6^-10^-3^] |  | [2000-2500] | [200-300] | [0.4-0.6] | [0.02-0.04] |  | [500-1000] | 5 | Tbot_1_ - 50 | 0.5 |
| 1Bott_1Recov_T_500_t_500_s_0.05 | [400-5000] | [10^-6^-10^-3^] |  | [2000-2500] | [200-300] | [0.4-0.6] | [0.02-0.04] |  | [500-1000] | 5 | Tbot_1_ - 500 | 0.05 |
| 1Bott_1Recov_T_500_t_500_s_0.2 | [400-5000] | [10^-6^-10^-3^] |  | [2000-2500] | [200-300] | [0.4-0.6] | [0.02-0.04] |  | [500-1000] | 5 | Tbot_1_ - 500 | 0.2 |
| 1Bott_1Recov_T_500_t_500_s_0.5 | [400-5000] | [10^-6^-10^-3^] |  | [2000-2500] | [200-300] | [0.4-0.6] | [0.02-0.04] |  | [500-1000] | 5 | Tbot_1_ - 500 | 0.5 |

Times are in generations, and migration rates in proportion of migrants per generation. Parameters are the time of divergence *Tdiv* between AGR and PYG, the migration rate *m* between AGR and PYG, the time *Texp_1_* of the first AGR instantaneous expansion and its growth rate *r_1_* (*N_Texp1_ = N_Texp1-1_ / r_1_*), the time *Texp_2_* of the second AGR exponential expansion and its exponential growth rate *r_2_*, the time *Tbot_1_* of the first PYG bottleneck and its strength *Sbot_1_* (*N_Tbot1_ = N_Tbot1-1_ / Sbot_1_*), the time *Trec_1_* of the first PYG recovery and its strength *Srec_1_* (*N_Trec1_ = N_Trec1-1_ / Srec_1_*). Parameters can (i) be constant (a single value is given; e.g. *Sbot_1_* is constant and equals 5 in 1Bott_1Recov models), (ii) have a flat prior distribution (an interval of two numbers in brackets is given; e.g. in the “Constant” model, *Tdiv* = [400-5000] means that *Tdiv* has a flat prior distribution ranging from 400 to 5000 generations) and (iii) be constrained by another parameter (e.g. in the 1Bott_1Recov_T_10_t_5_s_0.05 model, *Trec_1_* = *Tbot_1_*+5 means that *Trec_1_* is equal 5 plus the value of *Tbot_1_* retrieved from the flat prior distribution [10-100]).
