# Supplementary material for: Inferring the Demographic History of African Farmers and Pygmy Hunter–Gatherers Using a Multilocus Resequencing Data Set
Source: PLoS Genet. 2009 Apr 10;5(4):e1000448. doi: 10.1371/journal.pgen.1000448 (PMC2661362; doi:10.1371/journal.pgen.1000448)
Supplement: Table S7 — Prior distributions of the parameters of the IM models simulated to assess the branching history of the AGR, WPYG and EPYG populations, using the composite population dataset. (0.04 MB DOC) [file pgen.1000448.s012.doc]

**Table S7.** Prior distributions of the parameters of the IM models simulated to assess the branching history of the AGR, WPYG and EPYG populations, using the composite population dataset

|  | Model *W-AE* | Model *E-AW* | Model *A-WE* | Model *AWE* |
| --- | --- | --- | --- | --- |
| IM  parameters |  |  |  |  |
| *Tdiv_AGR-WPYG_* | *Tdiv_AGR-EPYG_*+[100-2500] | [301-2500] | *Tdiv_WPYG-EPYG_*+[100-2500] | [301-5000] |
| *Tdiv_AGR-EPYG_* | [301-2500] | *Tdiv_AGR-WPYG_*+[100-2500] | *Tdiv_WPYG-EPYG_*+[100-2500] | [301-5000] |
| *Tdiv_WPYG-EPYG_* | *Tdiv_AGR-EPYG_*+[100-2500] | *Tdiv_AGR-WPYG_*+[100-2500] | [301-2500] | [301-5000] |
| *m_AGR-WPYG_* | [10^-8^-10^-3^] | [10^-8^-10^-3^] | [10^-8^-10^-3^] | [10^-8^-10^-3^] |
| *m_AGR-EPYG_* | [10^-8^-10^-3^] | [10^-8^-10^-3^] | [10^-8^-10^-3^] | [10^-8^-10^-3^] |
| *m_WPYG-EPYG_* | [10^-8^-10^-3^] | [10^-8^-10^-3^] | [10^-8^-10^-3^] | [10^-8^-10^-3^] |
| Demographic  parameters |  |  |  |  |
| *Texp_1_* | *Tdiv_AGR-WPYG_*+5 | *Tdiv_AGR-EPYG_*+5 | *Tdiv_AGR-EPYG_*+5 | *Tdiv_AGR-EPYG_*+5 |
| *r_1_* | [0.6-0.8] | [0.6-0.8] | [0.6-0.8] | [0.6-0.8] |
| *Texp_2_* | [200-300] | [200-300] | [200-300] | [200-300] |
| *r_2_* | [0.02-0.05] | [0.02-0.05] | [0.02-0.05] | [0.02-0.05] |
| *Tbot_WPYG_* | [10-300] | [10-300] | [10-300] | [10-300] |
| *Sbot_WPYG_* | 5 | 5 | 5 | 5 |
| *Trec_WPYG_* | *Tbot_WPYG_*-5 | *Tbot_WPYG_*-5 | *Tbot_WPYG_*-5 | *Tbot_WPYG_*-5 |
| *Srec_WPYG_* | [0.2-1] | [0.2-1] | [0.2-1] | [0.2-1] |

Parameters are the times of divergence *Tdiv* between population pairs (in generations) and the migration rates *m* per population pairs (in proportion of migrants per generation). For the description of demographic parameters, see Table S6. Parameters can (i) be constant (a single value is given), (ii) have a flat prior distribution (an interval of two numbers in brackets is given) and (iii) be constrained by another parameter (e.g. in the *W-AE* model, *Tdiv_AGR-WPYG_=Tdiv_AGR-EPYG_*+[100-2500] means that *Tdiv_AGR-WPYG_* is equal to a random number between 100 and 2500 plus the *Tdiv_AGR-EPYG_* value randomly drawn from its prior distribution [301-2500]).
