# Supplementary material for: Inferring the Demographic History of African Farmers and Pygmy Hunter–Gatherers Using a Multilocus Resequencing Data Set
Source: PLoS Genet. 2009 Apr 10;5(4):e1000448. doi: 10.1371/journal.pgen.1000448 (PMC2661362; doi:10.1371/journal.pgen.1000448)
Supplement: Table S10 — Prior distributions and means of mutation rates and effective population sizes used for all coalescent simulations. (0.03 MB DOC) [file pgen.1000448.s015.doc]

**Table S10.** Prior distributions and means of mutation rates and effective population sizes used for all coalescent simulations

| Markers | *μ* distribution | *μ* mean | *N*_e_ distribution | *N*_e_ mean |
| --- | --- | --- | --- | --- |
| Autosomal | γ(2, 12.5) / 10^-9^ | 2.5x10^-8^ | γ(5, 2000) | 10,000 |
| X-linked | γ(2, 9) / 10^-9^ | 1.8x10^-8^ | γ(5, 2000) x 3 / 4 | 7,500 |
| Y-linked | γ(2, 13) / 10^-9^ | 2.6x10^-8^ | γ(5, 2000) / 4 | 2,500 |
| mtDNA | γ(2, 4.4) / 10-7 | 8.7x10^-7^ | γ(5, 2000) / 4 | 2,500 |

Effective population sizes *N_e_* are given in individuals and the mutation rate *μ* is given per site per generation. *N*_e_ and *μ* are drawn from γ distributions.
