# Supplementary material for: Inferring the Demographic History of African Farmers and Pygmy Hunter–Gatherers Using a Multilocus Resequencing Data Set
Source: PLoS Genet. 2009 Apr 10;5(4):e1000448. doi: 10.1371/journal.pgen.1000448 (PMC2661362; doi:10.1371/journal.pgen.1000448)
Supplement: Text S1 — Rationale of the study and supplementary analyses. (0.08 MB DOC) [file pgen.1000448.s016.doc]

**Inferring the Demographic History of African Farmers and Pygmy Hunter-Gatherers Using a Multilocus Resequencing Data Set**

**Rationale of the Study**

The ultimate goal of this study was to establish the branching history of Pygmy hunter–gatherer and agricultural populations from Africa, and to coestimate separation times and levels of gene flow between these groups of populations. We had to take several points into account to achieve this objective:

The use of pooled population groups*.* Our sampling scheme was designed to be representative of several populations of Pygmies and farmers. We tried to avoid inflation of the number of parameters to be estimated, by pooling our studied populations into three ethnologically recognized population groups: the Western Pygmy (WPYG), the Eastern Pygmy (EPYG) and the agricultural (AGR) groups, which also corresponded to the genetic clusters obtained with STRUCTURE for *K*=3 (Figure 2A). However, some populations within each of these three population groups showed substantial levels of admixture (Bakola and Twa Pygmies) or genetic differentiation (Mozambicans *vs*. other AGR populations). As a result, our pooling of populations into three groups may affect inferences concerning demographic regimes, branching history and/or the estimation of population separation times and gene flow levels. We explored these effects on our inferences, by systematically performing all analyses on two population datasets in parallel: (i) the entire population dataset, which included the admixed/structured individuals/populations (the “composite population dataset”) and (ii) a pruned population dataset that excluded individuals with ancestry in other populations, or populations that appeared to be differentiated at *K*=4 within each population group (the “filtered population dataset”). We excluded from this filtered dataset all individuals whose proportion of ancestry in another population group was higher than 20% at *K*=4, the most probable value of *K*. Using this filtering procedure, we excluded 92 individuals, including 15 Bakola, 2 C. Baka, 2 G. Baka, 4 Biaka, 1 Mbuti and 21 Twa Pygmies, as well as 4 Yoruba, 5 Ngumba, 5 Akele, 12 Chagga and 21 Mozambican farmers (Figure 2B).

The assessment of demographic regimes of each population group*.* Departures from nonequilibrium demography (e.g., population growth or shrinkage) have been shown to influence the estimation of population divergence and levels of gene flow [1,2]. We therefore aimed to consider the demographic regime of each population group for subsequent analyses. In this study, assessment of the internal demographic history of each population group was not a goal in itself and did not necessarily reflect the precise demographic regime characterizing these groups. Instead, the demographic models we used for each population group correspond to the simplest models best fitting the observed data and represent a prerequisite for confident inferences of divergence times and gene flow levels between our populations.

The use of an approximate Bayesian computation (ABC) approach. The likelihood function of complex demographic scenarios may rapidly become impossible to solve analytically, particularly when the number of parameters to be estimated is large [3]. Given the complexity of the historical and demographic models we aim to explore, we sought to overcome this problem by using a simulation-based procedure forming the basis of the ABC approach [4]. ABC approaches bypass the computational difficulties of using explicit likelihood functions by simulating data from a coalescent model. These methods rely on the simulation of large numbers of datasets using parameter values sampled from prior distributions. A set of summary statistics is then calculated for each simulated sample, and each set of simulated summaries is then compared with the values observed in the empirical data. Parameter values generating summary statistics similar enough to those of the empirical data are retained, allowing model choice and parameter estimation.

**Analyses Using the Composite Population Dataset**

We investigated the internal demographic regime of each population group and their branching history, and estimated the population separation times and levels of gene flow between populations, using the composite population dataset, which corresponds to the entire collection of unrelated samples.

Demographic Regimes of African Farmers and Pygmy Hunter-Gatherers*.* As already explained, the inference of divergence times and levels of gene flow between our population groups required an adjustment for the demographic regime of each population group. Here, we aimed to obtain the simplest demographic models best fitting the within-population statistics of each population group. A large proportion of the diversity of AGR populations was due to low-frequency variants (Figure S1), as attested by the high *θ*_W_ values obtained (Table S3). The excess of low-frequency variants was clearly supported by highly significant negative values for Tajima’s *D*, Fu & Li’s *D** and Fu’s *Fs* for autosomes, the Y chromosome and mtDNA (Table S3). The variance of Tajima’s *D* was also significantly lower across autosomal regions in the AGR group (Table S4). These observations strongly suggest the occurrence of population growth among the ancestors of present-day farmers. However, fine-scale population structure can also increase the proportion of singletons, when the studied sample is composed of several samples of weakly differentiated populations [5], which is the case in our composite sample of AGR. The signature of population growth as detected by neutrality tests was less pronounced but still significant when restricting the analyses to the filtered population dataset (see main text, Figure 3 and Table 1). Consequently, although fine-scale structure may have inflated the signal of growth observed for the AGR group in the composite population dataset, our data are consistent with population expansion in the AGR group.

None of the classical neutrality tests used detected significant departures from the constant-sized population model for the WPYG and EPYG groups (Table S3). However, the occurrence of gene flow between populations with different demographic regimes may dilute the signals of departure from nonequilibrium demography detected by neutrality tests. We investigated whether a constant-size population model was sufficient to account for the patterns of autosomal diversity observed for the WPYG and EPYG groups in the presence of various levels of gene flow with an expanding AGR population. We compared the empirical summary statistics obtained for the WPYG and EPYG population groups (Table S3) with summary statistics for 3,000,000 simulations, considering models of a constant-size population or bottlenecks varying in intensity, timing and duration (Figure S2, Table S6). We considered the existence of various levels of gene flow between this population and an expanding population with mean summary statistics similar to those for the AGR population group (Table S3). We simulated an AGR group experiencing an early instantaneous population expansion followed by a recent exponential expansion (Table S6) to obtain summary statistics (*S*, *π*, Tajima’s *D* and Fu & Li’s *D**) similar to our AGR data (Table S3). For the WPYG group, a bottleneck starting 250-2,500 years ago leading to an 80% decrease in population size, followed by a recovery starting 125 years later and involving an increase in population size of 0% to 100% (Figure S2), fitted the data significantly better than a constant-size model (*P*=0.02). For the EPYG group, a constant population size model without gene flow with AGR or models assuming a bottleneck followed by strong recovery (Figure S2) best fitted the data. However, it was not possible statistically to distinguish between the constant population size and bottleneck models (*P*=0.56).

Branching model and estimation of population separation times and gene flow. The specific demographic regime of each population group may influence the inference of their branching history [1,2]. We therefore incorporated into our simulations the demographic model for each population group most compatible with the observed within-population summary statistics (Table S3). We considered a model of population expansions for the AGR group, a model of bottleneck with recovery for the WPYG group, and a model of constant population size for the EPYG group (Table S7). Twenty autosomal regions were simulated 1,000,000 times under the four possible models of isolation-with-migration (IM) (Figure 5) with IM parameters (separation times, migration rates) drawn from large, flat prior distributions (Table S7). Independently of the set of summary statistics used, the *A-WE* model always gave the highest proportion of small distances between the simulated and observed datasets, and was therefore considered the most probable model, given the data (Figure S3). We then investigated the time scale of the events characterizing the branching history of the AGR, WPYG and EPYG populations, by estimating IM parameters under the validated *A-WE* model. Our estimations indicated that the ancestral effective population size of the African groups studied here was 9,428 individuals (95% CI: 6,791-15,151) (Table 2). This ancestral population pool started to diverge, eventually giving rise to the current-day agricultural and Pygmy populations, 60 Kya (95% CI: 25.2-120.1). The subsequent splitting of the ancestors of Pygmies into the WPYG and EPYG groups was estimated to have occurred 24.6 Kya (95% CI: 10.1-62.4). Finally, we estimated the levels of gene flow between WPYG and EPYG, between WPYG and AGR and between EPYG and AGR populations at 5.8x10^-4^, 2.2x10^-4^ and 6.6x10^-5^, respectively (Table 2). Remarkably, our estimates of population separation times were very similar for the composite and filtered population datasets (Table 2). In addition, our estimates of gene flow between population groups were consistently lower when we restricted the analyses to the filtered population dataset, which excludes admixed individuals/populations.

**Population Structure within the Western Pygmy Group**

Population structure within the WPYG group could mimic the detected effect of a bottleneck in this group (Figure 4). Indeed, significant differentiation was observed between WPYG populations (Table S2). We excluded this possibility by simply calculating the mean neutrality statistics for each WPYG population individually, across the 20 autosomal regions. We showed that the mean Tajima’s *D* and Fu & Li’s *D** values were all positive in each population group, with the exception of the Bakola, who displayed considerable admixture with the expanding AGR population (T*D* and *D** equal to 0.142 and 0.354 in the Biaka, 0.168 and 0.077 in the C. Baka, 0.029 and 0.183 in the G. Baka and -0.120 and -0.028 in the Bakola). As all WPYG populations have probably received migrants from expanding AGR populations [6], their positive neutrality statistics strongly suggest that a bottleneck occurred in their ancestral population, or alternatively, in each of the WPYG populations separately.

**Testing the IMa and *mimar* Programs**

We estimated divergence and gene flow between the AGR, WPYG and EPYG groups by two methods implemented in (i) the IMa program, which uses Monte-Carlo Markov chain (MCMC) simulations and analytical integrations to estimate parameters under an IM model [7], and (ii) the *mimar* program, which uses MCMC simulations to explore the posterior distribution of the IM parameters, given certain summary statistics for the data [8]. Both programs assume a two-population model, which may be problematic in our case study, given the non negligible levels of estimated gene flow between the three population groups (Figures 6 and S3, Table 2).

The IMa program assumes selective neutrality, no recombination within regions and free recombination between regions. Our autosomal dataset conforms well to these assumptions, because all 20 autosomal regions are far from any known gene, physically independent of each other, and short enough (~1.3 kb) to display very few recombinant haplotypes (~0.5% of haplotypes, which were excluded from this analysis). Five independent runs were performed for each population pair (the current version of IMa is based on a two-population IM model). Each run included 40 independent Markov chains with 780 chain swap attempts per step, 1,000,000 burnin steps and 10,000,000 trees to be saved during the MCMC. Estimations of parameters across runs were not concordant and ESS values, a measure of chain mixing, remained low (<50) despite millions of steps in parallel Markov chains (data not shown). Moreover, most posterior distributions were flat for migration rates. This probably indicates low convergence of the sample to stationary distributions (or insufficient running time) or may reflect insufficient power of IMa to estimate parameters when genetic differentiation is weak (IMa is partly based on fixed differences between populations or species, which do not exist between our three population groups).

The *mimar* program assumes selective neutrality and free recombination between regions, two conditions met by our autosomal dataset. Two independent runs were performed for each population pair (the current version of *mimar* is based on a two-population IM model). Each Markov chain included 5,500,000 steps, with 500,000 burnin steps. The prior distributions used for all parameters were those used for the ABC approach, reported in Table S7. We controlled for good mixing, using the rate of accepted steps (which should be at least 5%) and the autocorrelations of parameter values across steps (which should fall to zero as the number of steps increases). Estimates of divergence times differed between independent runs, indicating no convergence: *T*_WPYG-AGR_ was estimated at 61 Kya (95% CI: 48-87 Kya) and 92 Kya (95% CI: 78-121 Kya), *T*_EPYG-AGR_ was estimated at 79 Kya (95% CI: 58-110 Kya) and 101 Kya (95% CI: 80-120 Kya) and *T*_WPYG-EPYG_ was estimated at 76 Kya (95% CI: 56-115 Kya) and 94 Kya (95% CI: 75-123 Kya). In addition, the prior distributions of migration rates were flat for all runs (data not shown). These problems were not due to poor mixing of the Markov chains, because acceptance rates always exceeded 20% and autocorrelations rapidly decreased to zero for all parameters and all runs (data not shown). This is most likely due to the very low levels of differentiation between the populations studied (the *mimar* program should not be applied to populations with no fixed differences between them [8]).

**Summary Statistics as a Function of Levels of Divergence and Gene Flow**

We explored summary statistics sensitive to levels of divergence and gene flow between two demes, by simulating two populations of constant size with various divergence times (20, 200, 2,000, 20,000 generations) and migration rates (0, 10^-5^, 10^-4^, 10^-3^, 10^-2^, 10^-1^, 5x10^-1^). We ran 10,000 simulations for each of the 28 models. We then calculated various statistics that should depend on levels of divergence and gene flow (data not shown) [9]. We finally selected several summary statistics that turned out to be informative to discriminate between the confounding effects of divergence and gene flow on genetic variation: the proportion of mutations shared between populations $\Sigma_{i,j}$, the proportion of low-frequency shared mutations $\Sigma_{i,j}^{0.05}$ (shared alleles at a frequency lower than 0.05), the mean frequency of shared mutations $\hat{p}_{sS}$, and pairwise *F*_ST_. All these statistics are highly sensitive to fluctuations in divergence and gene flow (Figure S5).

**Factors contributing to the high levels of mtDNA differentiation observed between the Western and Eastern Pygmy groups**

By contrast with the autosomal, X-linked and Y-linked regions, mtDNA displayed very high levels of differentiation between the two Pygmy groups, regardless of the population dataset considered (Table S5). Both groups were closer to the AGR group than to each other, consistent with the findings of previous studies [10,11]. The smaller effective population size for mtDNA than for autosomes (one quarter that of autosomes, due to mtDNA haploidy and uniparental inheritance) may account for this observation. Genetic drift has probably been strongest in hunter-gatherer groups, so Pygmy mtDNAs have been more severely affected by its stochastic effects (e.g., an increase in population differentiation) than autosomes. However, stronger genetic drift alone among Pygmies is insufficient to account for all our results. First, although the effective population sizes for the Y and X chromosomes are also smaller than that of the autosomes (one quarter and three quarters, respectively), the levels of population differentiation between Pygmies inferred from the sex chromosomes were low and very similar to those observed for autosomes (Table S5). Second, when we simulated an mtDNA locus (Materials and Methods) under the most probable *A-WE* model deduced from autosomal regions (Figures 6 and S3), the *F*_ST_ value between Pygmies was indeed the highest (mean simulated *F*_ST_ = 0.16 and 0.15 using the best-fitting models defined for the filtered and composite population datasets, respectively), but remained much lower than those observed in this study (observed *F*_ST_ = 0.38 and 0.39 for the filtered and composite population datasets, respectively; Table S5).

Sex-biased gene flow and ancient maternal population structure have probably contributed to the high levels of mtDNA differentiation observed today between the two Pygmy groups. Our data indicate a sex bias in migration events, with lower levels of maternal gene flow between the WPYG and EPYG groups than between the Pygmy groups and their neighboring agricultural populations. In addition, previous mtDNA data indicated that long-standing maternal gene flow has occurred mostly in the PYG-to-AGR direction [11]. However, male-biased gene flow in the opposite direction (i.e. from AGR-to-PYG) has been documented on the basis of Y-chromosome data [12,13]. The relative genetic isolation between WPYG and EPYG at the mtDNA level, together with the substantial male-specific contribution of AGR to PYG, may account for the low levels of differentiation between the two groups of Pygmies in all genetic compartments other than mtDNA. With respect to ancient population structure, the retention by genetic drift of different mtDNA lineages from a common ancestral, structured population of the WPYG and EPYG groups may have further exacerbated the observed levels of divergence between these groups at the mtDNA level. Given the deep coalescent times of the mtDNA clades characterizing these populations [14], the ancestral population ultimately giving rise to present-day Pygmies probably had an mtDNA gene pool composed of highly divergent lineages. Taken together, a number of non mutually exclusive factors may have contributed to the high level of maternal differentiation observed between the two Pygmy groups: strong genetic drift, sex-biased gene flow, periods of population shrinkage and the differential retention of divergent lineages from an already structured ancestral maternal gene pool.

**References**

1. Hey J (2005) On the number of New World founders: a population genetic portrait of the peopling of the Americas. PLoS Biol 3: e193.

2. Garrigan D, Kingan SB, Pilkington MM, Wilder JA, Cox MP, et al. (2007) Inferring human population sizes, divergence times and rates of gene flow from mitochondrial, X and Y chromosome resequencing data. Genetics 177: 2195-2207.

3. Marjoram P, Tavaré S (2006) Modern computational approaches for analysing molecular genetic variation data. Nat Rev Genet 7: 759-770.

4. Beaumont MA, Zhang W, Balding DJ (2002) Approximate Bayesian computation in population genetics. Genetics 162: 2025-2035.

5. Ptak SE, Przeworski M (2002) Evidence for population growth in humans is confounded by fine-scale population structure. Trends Genet 18: 559-563.

6. Verdu P, Austerlitz F, Estoup A, Vitalis R, Georges M, et al. (2009) Origins and genetic diversity of Pygmy hunter-gatherers from Western Central Africa. Curr Biol 19: 312-318.

7. Hey J, Nielsen R (2007) Integration within the Felsenstein equation for improved Markov chain Monte Carlo methods in population genetics. Proc Natl Acad Sci USA 104: 2785-2790.

8. Becquet C, Przeworski M (2007) A new approach to estimate parameters of speciation models with application to apes. Genome Res 17: 1505-1519.

9. Wakeley J (1996) Distinguishing migration from isolation using the variance of pairwise differences. Theor Popul Biol 49: 369-386.

10. Salas A, Richards M, De la Fe T, Lareu M, Sobrino B, et al. (2002) The making of the African mtDNA landscape. Am J Hum Genet 71: 1082-1111.

11. Quintana-Murci L, Quach H, Harmant C, Luca F, Massonnet B, et al. (2008) Maternal traces of deep common ancestry and asymmetric gene flow between Pygmy hunter-gatherers and Bantu-speaking farmers. Proc Natl Acad Sci USA 105: 1596-1601.

12. Cruciani F, Santolamazza P, Shen P, Macaulay V, Moral P, et al. (2002) A back migration from Asia to sub-Saharan Africa is supported by high-resolution analysis of human Y-chromosome haplotypes. Am J Hum Genet 70: 1197-1214.

13. Wood ET, Stover DA, Ehret C, Destro-Bisol G, Spedini G, et al. (2005) Contrasting patterns of Y chromosome and mtDNA variation in Africa: evidence for sex-biased demographic processes. Eur J Hum Genet 13: 867-876.

14. Behar DM, Villems R, Soodyall H, Blue-Smith J, Pereira L, et al. (2008) The dawn of human matrilineal diversity. Am J Hum Genet 82: 1130-1140.
